# Membrane translocation of folded proteins

**DOI:** 10.1016/j.jbc.2022.102107

**Published:** 2022-06-04

**Authors:** Dehua Pei, Ross E. Dalbey

**Affiliations:** Department of Chemistry and Biochemistry, The Ohio State University, Columbus, Ohio, USA

**Keywords:** protein translocation across membranes, folded proteins, twin arginine translocation, peroxisome protein import, mitochondrial protein import, endosomal escape, unconventional protein secretion, vesicle budding and collapse, AC, adenylate cyclase, CDT, *Clostridioides difficile* transferase toxin, CPP, cell penetrating peptides, DT, diphtheria toxin, ER, endoplasmic reticulum, GUV, giant unilamellar vesicle, LUV, large unilamellar vesicle, MVB, multivesicular bodies, PA, protective antigen, PI(4,5)P2, phosphatidyl-4,5-bisphosphate, Sec, secretory, TAT, twin-arginine translocation, T2SS, type 2 secretion system, T7SS, type 7 secretion system, T9SS, type 9 secretion system, TM, transmembrane, UPS, unconventional protein secretion, VBC, vesicle budding and collapse

## Abstract

An ever-increasing number of proteins have been shown to translocate across various membranes of bacterial as well as eukaryotic cells in their folded states as a part of physiological and/or pathophysiological processes. Herein, we provide an overview of the systems/processes that are established or likely to involve the membrane translocation of folded proteins, such as protein export by the twin-arginine translocation system in bacteria and chloroplasts, unconventional protein secretion and protein import into the peroxisome in eukaryotes, and the cytosolic entry of proteins (*e.g.*, bacterial toxins) and viruses into eukaryotes. We also discuss the various mechanistic models that have previously been proposed for the membrane translocation of folded proteins including pore/channel formation, local membrane disruption, membrane thinning, and transport by membrane vesicles. Finally, we introduce a newly discovered vesicular transport mechanism, vesicle budding and collapse, and present evidence that vesicle budding and collapse may represent a unifying mechanism that drives some (and potentially all) of folded protein translocation processes.

All prokaryotic and almost all eukaryotic proteins are synthesized by ribosomes inside the cytoplasm. During or soon after their synthesis, many of the proteins must be transported to specific subcellular locations or exported from the cell to exert biological functions. In eukaryotes, more than one third of the proteins are targeted to organelles, including endoplasmic reticulum (ER), peroxisomes, mitochondria, and plastids (1), while in bacteria it is estimated that ∼8% of all proteins are secreted to the periplasmic/extracellular space (2). In each of the aforementioned targeting events, the protein must travel across at least one and sometimes two or more cell membranes. It is now clear that proteins can be transported across cell membranes in either the unfolded or the folded state (Fig. 1). For protein translocation in the unfolded state, a well-studied system is the classical secretory (Sec) pathway, which is evolutionarily conserved and operates in the plasma membrane of bacteria, the ER membrane of eukaryotes, and the thylakoid membrane of chloroplasts in plants. Proteins containing hydrophobic leader sequences are bound by cytosolic chaperones/targeting factors and kept in their unfolded state. Subsequently, the unfolded polypeptide is transferred to a membrane-embedded protein channel (the translocon) and threaded through the channel in an energy-dependent process or laterally released into the lipid bilayer (3). Examples of protein-conducting channels include the Sec61 complex of the ER membrane (SecYEG in prokaryotes) and the mitochondrial TOM complex. For more information about the Sec pathway, readers are referred to several reviews (4, 5, 6).

**Figure 1:**
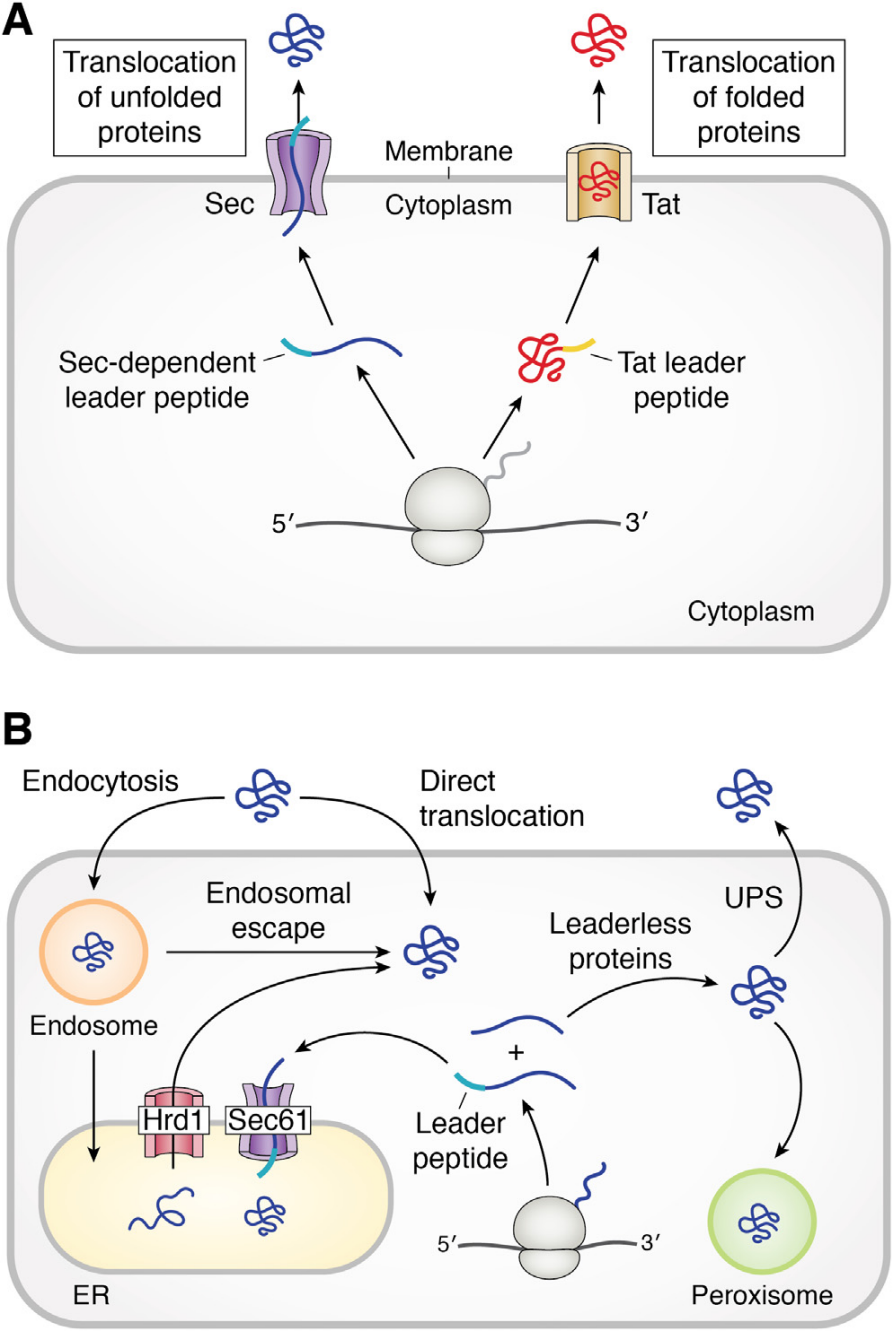
**Protein translocation across various cellular membranes by conventional and unconventional pathways.***A*, protein export by the Sec (unfolded proteins) and TAT pathways (folded proteins) in bacteria. The Sec-dependent leader peptide is shown in *cyan* and the Tat leader peptide in *yellow*. *B*, protein transport pathways in eukaryotic cells. Unfolded, leader peptide (shown in *cyan*)-containing proteins are transported into the ER by the Sec61 complex, whereas folded, leaderless proteins are secreted by the UPS pathway or imported into the peroxisome. Folded proteins also enter the cytosol of eukaryotic cells by crossing the plasma, endosomal, or ER membrane. Hrd1 forms a retrotranslocation channel. ER, endoplasmic reticulum; Sec, secretory; TAT, twin-arginine translocation; UPS, unconventional protein secretion.

Proteins and protein complexes can also move across cellular membranes in the folded state to reach their final destinations inside as well as outside the cell (Fig. 1). In bacteria and chloroplasts, folded proteins are transported across the plasma or thylakoid membrane by the twin-arginine translocation (TAT) system into the periplasmic/extracellular space and the thylakoid, respectively (7). In eukaryotes, folded proteins are transported across the plasma membrane (*i.e.*, from the cytosol to the extracellular environment) by several different mechanisms that have been collectively termed “the unconventional protein secretion (UPS) system” (8). Folded proteins and protein complexes are also imported into the subcellular organelles of eukaryotes (*e.g.*, peroxisomes) (9). In addition, certain viral, bacterial, and eukaryotic proteins enter the eukaryotic cell autonomously, by crossing the plasma, endosomal, or ER membrane (10). Recent data demonstrated that at least some of the latter proteins enter the cell in the folded state (11, 12, 13). How folded proteins move across the cell membrane has been a longstanding mystery in cell biology. One of the greatest enigmas is how folded proteins or protein complexes, which may have a diameter of >100 Å, cross a lipid bilayer without compromising the barrier function of the membrane. Equally perplexing is how the same translocation machinery (*e.g.*, the TAT system) accommodates protein substrates of varied sizes and different physicochemical properties. Understanding the mechanism of membrane trafficking by folded proteins will also have important applications in biotechnology, for example, the design of cell-permeable proteins as novel therapeutics.

This review focuses on the translocation of folded proteins across the sealed membranes of bacteria and eukaryotes in both directions (*i.e.*, protein export and import). We will first provide an updated summary of the systems involving membrane translocation of folded proteins (defined here as proteins that maintain their 3D structures throughout the membrane translocation process) and their biological functions. We will next discuss the various mechanistic hypotheses that have been put forth for the membrane translocation of folded proteins and any evidence for and/or against them. Finally, we present evidence that a recently discovered membrane transport mechanism (13), vesicle budding and collapse (VBC), may be a unifying mechanism that drives the membrane translocation of some (and potentially all) of the folded proteins.

## Folded proteins translocate across cell membranes

Once thought as rare and an exception to the rule, an increasing number of systems from all three domains of life have now been shown to translocate folded proteins across different cellular membranes. For systems that are well established (*e.g.*, the TAT system, the UPS system, and protein import into the peroxisome), we will only briefly introduce them. For systems that were recently discovered or are not yet firmly established, we will provide a more in-depth discussion including any evidence for protein translocation in the folded state.

### Established systems that transport folded proteins

#### TAT system

The TAT pathway represents the best characterized system for the transport of folded proteins in bacteria and plants (7, 14, 15, 16) (Table 1). Initially discovered in chloroplasts (17, 18, 19), the TAT system is present in most bacteria (20, 21, 22) and has also been identified in archaea (23), while many mitochondria have lost it during the evolution from their bacterial ancestor (24). Unlike the Sec pathway, proteins exported by the TAT pathway are characterized by having a twin-arginine signal sequence (25) and require typically the TatA, TatB, and TatC components (TatABC complex) or only TatA and TatC (TatAC) (22, 26). Many of the 30 TAT substrate proteins in *Escherichia coli* contain complex metal cofactors such as Fe-S clusters and molybdopterin centers (25, 27). These proteins are fully folded prior to export. Further, there exist quality control systems that ensure the proper folding of many of the TAT substrates, with unfolded substrates rejected for export (27). Translocation of proteins in their folded states avoids the need to reassemble complex cofactors in the extracellular environment.

**Table 1:** Characteristics of membrane translocation systems

Translocation system	Signal sequence?	Substrate folded?	Reference
ER and bacterial Sec	N-terminal	No	Joly and Wickner (195); van den Berg *et al.* (196)
ER to cytosol	None	?	Inoue and Tsai (111)
Bacterial and chloroplast TAT	N-terminal	Yes	Palmer *et al*. (27); Albiniak *et al.* (197)
UPS (Type I)	None	Yes	Rabouille *et al.* (198)
UPS (Type II)	None	Yes	Rabouille *et al.* (198)
UPS (Type III)	None	Yes	Rabouille *et al.* (198)
Cytosol to peroxisome	N- or C-terminal	Yes	Leon *et al.* (9); Yang *et al.* (58)
Mitochondrial TOM	N-terminal	No	Eilers and Schatz (199)
Mitochondrial Bcs1 AAA	None	Yes	Wagener *et al.* (112)
Chloroplast TOC	N-terminal	Partially	Ganesan *et al.* (200)
Bacterial T2SS	N-terminal	Yes	Hirst and Holmgren (63); Pugsley *et al.* (64)
Bacterial T7SS	C-terminal	Yes	Bowman and Palmer (201)
Bacterial T9SS	N- and C-terminal	Yes	Lauber *et al.* (118)
Endosomal escape	None	Yes	Sahni and Pei (13)
Cell entry by direct translocation	None	Yes	Hariton-Gazal *et al.* (92); Williams and Tsai (10)

#### UPS pathways

Some eukaryotic proteins do not contain any leader sequence and are yet exported into the extracellular environment by several different mechanisms that have been collectively referred to as the “UPS” pathways (Fig. 1*B*) (28, 29, 30). UPS is often induced by cellular stresses (*e.g.*, nutrient starvation (31)) or other environmental cues (*e.g.*, infection by pathogens (32)). A well-known example is interleukin-1β (IL-1β), a potent proinflammatory cytokine critical for host response to infection, while excessive secretion of IL-1β leads to a myriad of human diseases (32). Other prominent examples include fibroblast growth factors 1 (FGF1) and 2 (FGF2) (33, 34), annexins (35), galectins (35), acyl-CoA–binding proteins (AcbA and Acb1) (31, 36, 37, 38, 39, 40), HIV-1 transactivator of transcription (HIV-Tat) (41), Tau (42) and various enzymes (*e.g.*, phosphoglycerate kinase 1 (43)). A comprehensive list of proteins known to undergo UPS can be found in several recent reviews (29, 30, 44). Most of these proteins are derived from higher eukaryotes (45); however, UPS has also been observed in bacteria (46). There is compelling evidence that during UPS, proteins move across the plasma membrane in their folded states (47, 48).

#### Protein import into peroxisomes

Unlike protein transport into other organelles such as the ER, mitochondria, and chloroplasts, proteins imported into the peroxisome cross the membrane barrier in the folded states (Fig. 1*B*) (9, 49). Protein targeting to the peroxisome is directed by a C-terminal signal sequence of the consensus (S/A/C)-(K/R/H)-L (PTS1) (50, 51) or an N-terminal sequence of R-(L/V/I/Q-X_2_-(L/V/I/H)-(L/S/G/A)-X-(H/Q)-(L/A) (PTS2) (52), which are recognized by the Pex5 and Pex7 receptors, respectively. An early indication that proteins are imported in a folded state originated from peroxisomal import studies of catalase in fibroblasts, demonstrating that the metal cofactor-containing enzyme is imported as an oligomer (53). Later microinjection studies in mammalian cells showed that a folded luciferase, an octameric alcohol oxidase, and the heteropentameric acyl-CoA oxidase are imported into peroxisomes (54, 55, 56). Definitive evidence for the import of folded proteins into the peroxisome came from “piggyback” transport experiments, during which multisubunit protein complexes were imported into the peroxisome when only one of the subunits contains a PTS motif (57). Even nonprotein cargos such as DNA (58), polysaccharides (58), and gold nanoparticles of up to 90 Å in diameter (59) have been imported into the peroxisome by the “piggyback” mechanism. To date, the largest entity that has been imported into the peroxisome is a mCherry oligomer with a molecular weight of 619 kDa and a diameter of 126 Å (58).

### Systems that likely transport folded proteins

#### Secretion of bacterial proteins

Bacterial cells utilize at least nine different types of secretion systems to export protein toxins, degradative enzymes, adhesins, and other exoproteins into the extracellular medium, but the molecular mechanisms of these systems remain incompletely understood (60). There is compelling evidence that several of the secretion systems involve translocation of folded proteins, especially across the outer membrane of Gram-negative bacteria. This was first demonstrated for the type 2 secretion system (T2SS), in which proteins are first exported to the periplasm, usually by the Sec pathway but also by the TAT pathway; as a second step, the proteins then move across the outer membrane and into the extracellular environment (61, 62). Hirst and Holmgren showed that the fully assembled cholera toxin (which consists of an A subunit bound to a ring of five B subunits) was secreted by *Vibrio cholerae* (63). Both A and B subunits are synthesized in a precursor form and exported into the periplasm by the Sec pathway. While inside the periplasm, the subunits assemble into the AB_5_ oligomer (88 kDa) with a half-life of ∼1 min. Subsequently, the AB_5_ oligomer is transported across the outer membrane as a complex with a half-life of ∼13 min. Other proteins secreted by T2SS in the folded states include pullulanase, cellulase, pectate lyase, and the dimeric proaerolysin (64, 65, 66, 67, 68). Some of the secreted proteins form disulfide bonds while inside the periplasm (64, 65). How the T2SS machinery recognizes substrates is not known, but the involvement of linear secretion signal(s) has been excluded. Current data suggest that the secretion signal may consist of noncontiguous epitopes within the folded protein or protein complex.

Like T2SS, the type 9 secretion system (T9SS) also transports proteins first to the periplasm and then moves them across the outer membrane in a folded state (69, 70). It is capable of secreting exceptionally large proteins (*e.g.*, SprB, which is 6497 aa in length). T9SS substrates possess an N-terminal signal peptide which is recognized by the Sec machinery and require a folded C-terminal domain for export (71, 72). The type 7 secretion system of mycobacteria appears to export folded proteins across the plasma membrane and their unusual outer membrane (the so-called “mycomembrane”) in a single step. EsxA and EsxB of *Mycobacterium tuberculosis* are small proteins with a helix-turn-helix structure and form an obligatory heterodimer *in vivo*. Although only one of the monomers (EsxB) contains a secretion signal, both monomers are secreted, apparently as a heterodimeric complex, indicating that they are secreted in the folded state (73, 74, 75, 76).

#### Endosomal escape of bacterial toxins

Bacterial protein toxins reach the cytosol of eukaryotic host cells by crossing the plasma, endosomal, or ER membrane (Fig. 1*B*). AB toxins, which are most common and best characterized, typically consist of two functional units, an enzymatic A moiety and a nonenzymatic B moiety, which mediates receptor binding (R-domain) and membrane translocation (T-domain). For AB toxins that enter the host cell by endocytosis, it is commonly believed that the T-domain undergoes a conformational change upon endosomal acidification and inserts into the endosomal membrane to form a pore/channel, and the unfolded A moiety threads through the pore/channel *via* a charge state–dependent Brownian ratchet mechanism (77). Biochemical and electrophysiological studies have provided conclusive evidence for the formation of an ion-conducting channel in the endosomal membrane by different bacterial toxins including diphtheria toxin (DT) (78), *Bacillus anthracis* protective antigen (PA) (79), and the *Clostridioides difficile* transferase toxin (CDT) (80). Recent cryo-EM structures of PA and CDT show that seven copies of the toxins assemble to form a membrane-spanning 14-stranded β-barrel 93 to 105 Å in length and 27 Å (from Cα to Cα) in diameter (81). The mouth of the channel has a 30 Å opening, while the main body of the channel has inner diameters of 12 to 18 Å and is rich in hydrophilic residues. This suggests that the channel can accommodate unfolded polypeptides and perhaps protein secondary structure elements but not folded domains. A Φ-clamp formed by phenylalanine residues (Phe-427 in PA) just below the mouth becomes the bottleneck of the entire channel, with a solvent-excluded inner diameter of only 6 Å, which is smaller than protein secondary structure elements and therefore may only allow passage of fully unfolded polypeptides.

Although PA and CDT channels have the proper dimensions to accommodate unfolded polypeptides, to our knowledge, direct evidence that proteins (either folded or unfolded) pass through the channel is not yet available. On the other hand, there is compelling evidence that at least some bacterial toxins escape the endosome in the folded state. For example, DT is capable of delivering hyperstable cargo proteins (11) as well as noncovalently associated nucleic acids (82, 83) into the cytosol of mammalian cells. This indicates that DT moves across the endosomal membrane in the folded state, as unfolding of DT inside the endosome would result in the dissociation of the noncovalently attached cargo and failure to deliver the nucleic acids into the cytosol. NleC is a Zn^2+^ metalloprotease produced by pathogenic *E. coli* and consists of a single catalytic domain of 330 residues (84). NleC requires its intact native structure for host cell entry, since NleC mutants with altered 3D structures were defective in cell entry (12).

#### Cellular entry of viruses

While enveloped viruses are bound by a lipid bilayer allowing them to enter host cells by membrane fusion, nonenveloped viruses are surrounded by a proteinaceous capsid and must rely on viral proteins to gain host cell entry. Three major types of cell entry modalities have been found in the “membranolytic” viral proteins: amphipathic α-helical domains (*e.g.*, adenovirus protein VI (85)), myristoylated proteins (*e.g.*, N-myristoylated capsid protein μ1 of reovirus (86)), and membrane-remodeling enzymatic domains (*e.g.*, the phospholipase A type 2 domain of parvovirus VP1 (87)). These modalities allow nonenveloped viruses to enter the cytosol by moving across the endosomal (*e.g.*, adenovirus (88)), the Golgi (*e.g.*, papillomavirus (89)), or the ER membrane (*e.g*., SV40 (90, 91)). Their ability to travel across the lipid bilayer has often been recapitulated with artificial membrane vesicles (85, 86). How these structurally different modalities mediate the membrane translocation of a mostly intact viral particle remains unclear. It is clear, however, that unfolding of the viral proteins would result in the disassembly of the viral particle and block the cytosolic/nuclear entry of the viral genome.

#### Cellular entry of eukaryotic proteins

Eukaryotic proteins have also been found to enter eukaryotic cells autonomously by traversing the plasma or endosomal membrane (Fig. 1*B*). For example, human histone proteins (H2A, H2B, H3, and H4) directly cross the plasma membrane and deliver macromolecules covalently attached to them into cultured HeLa and Colo-205 cells (92). Cytosolic entry occurred under conditions that block the endocytic pathway, for example, at 4 °C, in ATP-depleted cells, in cells incubated with sucrose (0.5 M), or in the presence of various endocytosis inhibitors. Cell-to-cell transmission of α-synuclein is implicated in the progression of Parkinson’s disease; it is released by diseased cells to the extracellular fluid and is subsequently taken up by healthy cells nearby through endocytic mechanisms (93). Similarly, a splicing variant of protein and lipid phosphatase PTEN, PTEN-long, is secreted by donor cells and later enters recipient cells to regulate PI3K signaling in the latter cells (94). Finally, cell-permeable autoantibodies against nuclear DNA were discovered in patients with lupus (95). The mechanisms of cell entry by these proteins have not yet been established; however, our survey of the literature suggests that they cross the plasma or endosomal membrane in the folded state.

#### Cellular entry of engineered proteins

Cell-permeable proteins have been engineered as research tools and novel therapeutics. Earlier researchers took advantage of the modular structures of bacterial toxins to design cell-permeable chimeric proteins by either replacing their receptor-binding domains with proteins that bind selectively to the surface of cancer cells or fusing their membrane-translocation domains with exogenous cargo proteins. These efforts led to several immunotoxin drugs for cancer treatment (96). More recent efforts involved the introduction of cell-penetrating motifs into human proteins. A widespread practice is to genetically fuse a short cell-penetrating peptide (CPP) sequence (*e.g.*, Tat, R9, or penetratin) to the N or C terminus of a protein of interest (97). Proteins (*e.g.*, protein-tyrosine phosphatase 1B) may also be rendered cell-permeable by grafting a short CPP sequence (*e.g.*, RRRRWWW) into one of their surface loops (98). Finally, inspired by Lupus-derived autoantibodies (95), Kim *et al*. engineered cell-permeable antibodies against challenging intracellular targets as novel anticancer agents (99). The cell entry mechanisms of the engineered proteins remain unknown; the available data suggest that they enter the mammalian cell as folded proteins (98) (Fig. 1*B*).

#### Retrograde protein transport in the ER

The ER possesses a quality control system, the ER-associated degradation pathway, which ensures a misfolded protein in the ER is transported back into the cytosol for degradation by the proteasome (Fig. 1*B*) (100, 101, 102, 103). Some bacterial and plant AB toxins enter the host cell by endocytosis and make their way into the ER where the complex disassembles, and the enzymatically active A subunit is thought to hijack the ER-associated degradation apparatus to retrotranslocate into the cytosol (104). Examples include cholera toxin, Shiga toxin, ricin, *Pseudomonas* exotoxin PE, Pertussis toxin, and cytolethal distending toxins (105). It has been reported that the A subunits of these toxins are transported across the ER membrane in their partially unfolded state (106) by the Hrd1 complex. However, some nonenveloped viruses, for example, Polyoma and SV40 viruses (107, 108, 109, 110), also enter the cytosol of host cells by way of the ER. Since the viral particles in the ER are largely intact and have dimensions of 400 to 500 Å (111), the latter observation argues that the ER membrane may contain a system, possibly the ER retrograde apparatus in some cases, which transports folded proteins.

#### Protein translocation in mitochondria

During evolution, a novel pathway arose within the mitochondria for translocation of a folded Fe-S protein from the mitochondrial matrix to the intermembrane space compartment (112). While in bacteria, Fe-S proteins are exported by the TAT pathway (15, 27), the TAT pathway has been largely lost from most fungi and animal mitochondria. Therefore, an alternative pathway is needed to transport folded proteins. The transport of the Fe-S protein Rip1 is required for the assembly of the bc1 respiratory complex in the inner membrane of mitochondria. Rip1 is a nuclearly encoded protein that is synthesized in a precursor form with a mitochondrion-targeting sequence and is imported into the matrix in an unfolded state by the TOM and TIM23 complex. While inside the matrix, a 2Fe-2S cluster is inserted into the polypeptide to form the folded, globular C-terminal domain, which is then transported across the inner membrane, while the N-terminal transmembrane segment is imbedded into the inner membrane. Remarkably, the Bcs1 AAA ATPase catalyzes the translocation of the folded Fe-S domain of Rip1 that has an effective diameter of 25 Å.

## **Previously proposed mechanisms for translocation of foldedproteins**

Many mechanistic hypotheses have been put forth for the transport of folded proteins across different cell membranes, but few of them have been experimentally validated. These mechanisms can be classified into four major categories: pore/channel formation, local membrane disruption, membrane thinning, and transport by vesicles. The first three mechanisms involve a folded protein physically passing through a lipid bilayer, whereas the last does not. We will briefly describe how each mechanism works, its involvement to biologically relevant protein transport processes, and any evidence for as well as against it.

### Pore/channel formation

#### Bacterial outer membrane pores

**Figure 2:**
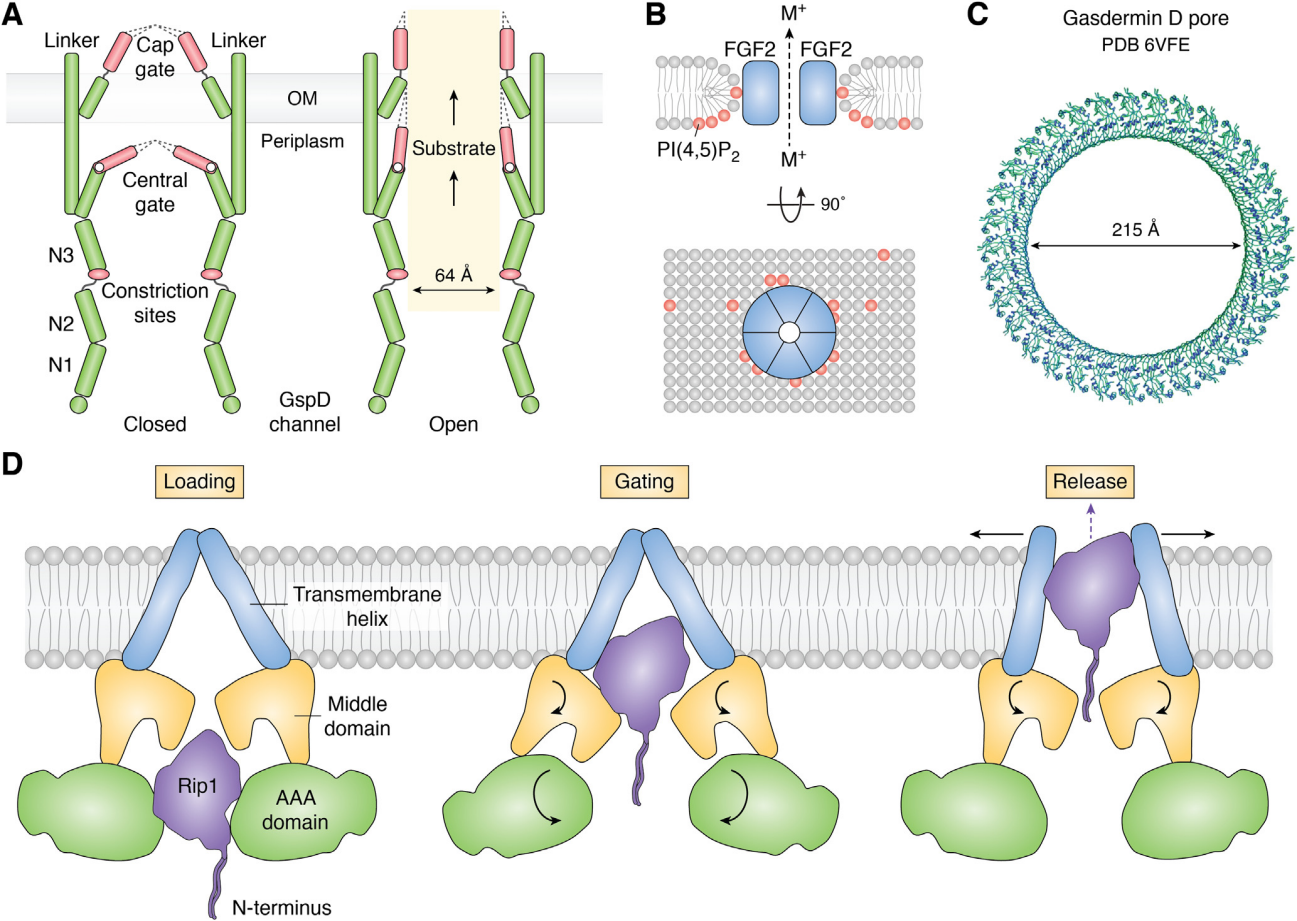
**Proposed mechanisms for translocation of folded proteins through pore/channel formation.***A*, the mechanism of gate opening of the GspD channel and a model of *Vibrio cholerae* GspD channel with a partially opened central gate (PDB 5WQ9). The N3 constriction sites, periplasmic central gate, and the extracellular gap gate open by the passage of the protein substrate. Adapted from Fig. 6 in (116). *B*, a lipidic toroidal pore formed by hexameric FGF2, which interacts with PI(4,5)P2 (represented by *red spheres*) in the inner leaflet of the plasma membrane (33). A small opening in the center allows small molecules and ions to move through. *C*, cryo-EM structure of the pore formed by gasdermin D (PDB 6VFE). *D*, model showing the transfer of a folded protein (Rip1) through an AAA membrane transporter. Adapted from Fig. 6 in (129). PDB, Protein Data Bank; PI(4,5)P2, phosphatidyl-4,5-bisphosphate.

The T2SS is a molecular machine that spans the inner and outer membranes of Gram-negative bacteria and comprises as many as 15 proteins (113, 114). It consists of an inner membrane platform with an associated cytoplasmic ATPase, a periplasmic pseudopilus, and a piston-shaped outer membrane secretion channel. Folded proteins cross the outer membrane *via* the secretion channel GspD (115). The structure of GspD has been solved by cryo-EM to high resolution and features a pentadecameric channel with 60 β-strands in each barrel (116) (Fig. 2*A*). The channel is gated on the periplasmic and extracellular sides by the central and cap gates. During transport of the cargo across the secretion channel, it is expected that the gates are wide open, with diameters of 53 Å and 48 Å at the periplasmic and extracellular sides, respectively (116). It was hypothesized that the pseudopili would contact the substrate in the periplasm and push the folded substrate through the secretion channel, thereby triggering the gates to open and even further expand the channel barrel (116, 117).

The T9SS SprA translocon, located in the outer membrane of Gram-negative bacteria, represents the largest monomeric β-barrel identified so far (36 β-strands) and transports folded proteins. Two structures of SprA have been solved, one bound with PorV and peptidyl-prolyl cis–trans isomerase (PPI) while the other with the Plug protein and PPI (118). One of the structures shows SprA open from the periplasmic side but the lateral opening is capped by PorV, a shuttle protein that recognizes cargo proteins through their conserved C-terminal domain signal (119, 120). In the other structure, the periplasmic side of the SprA channel is sealed by the plug protein and the channel is open to the extracellular side. The binding of PorV and Plug are mutually exclusive, and the channel opens alternately between the periplasmic or extracellular sides of the outer membrane through conformational changes within the barrel itself. This model suggests that in the SprA–PorV state, the substrate enters the SprA channel from the periplasm and binds to the PorV protein; after the PorV–substrate complex is released, the Plug protein seals the SprA channel from the periplasmic side until PorV binds again to the lateral opening of the channel.

#### Plasma membrane pores

Pore formation in the plasma membrane (type I UPS) has been hypothesized for the unconventional secretion of FGF2, HIV-Tat, annexins, and IL-1β/gasdermin D (29). It was proposed that FGF2 binds to negatively charged phosphatidyl-4,5-bisphosphate (PI(4,5)P2) on the inner plasma membrane and oligomerizes into a hexamer, which inserts into the membrane to form a transient lipidic pore with a toroidal architecture (Fig. 2*B*) (33). Upon emerging from the outer leaflet, the membrane-inserted oligomers disassemble, bind to heparan sulfates at the cell surface with high affinity (*K*_D_ = 10 nM), and are trapped at the cell surface. This model is supported by biochemical and biophysical evidence including high-affinity binding of FGF2 to PI(4,5)P2 (*K*_D_ = 1 μM), PI(4,5)P2-dependent oligomerization of FGF2 on membrane surface, and the PI(4,5)P2-dependent formation of “pores” on giant unilamellar vesicles (GUVs) that allowed small molecules (*e.g.*, Alexa488 dye) to move across the GUV membrane. The model explains the directional transport of FGF2 from the cytoplasm into the extracellular space and is consistent with the observation that membrane translocation requires FGF2 to be properly folded during all stages of this process. HIV-Tat was proposed to form a similar pore on the plasma membrane (121). The hypothetical pore formed by annexins has not been characterized.

IL-1β is mainly expressed in myeloid cells (*e.g.*, macrophages and monocytes) as a 31 kDa inactive form, pro-IL-1β. Activation of the inflammasome (*e.g.*, as a result of viral infection) recruits caspase-1, which subsequently converts pro-IL-1β into mature IL-1β by removing the N-terminal 117 residues (44). Removal of the highly acidic N terminus increases the pI of IL-1β from 4.6 to 8.8 and exposes a C-terminal polybasic sequence, ^88^KNYPKKK^94^, allowing mature IL-1β to relocate from the cytosol to plasma membrane microdomains, which are enriched in PI(4,5)P2 (122). In resting, nonpyroptotic myeloid cells, IL-1β is slowly released from these microdomains into the extracellular environment without compromising the integrity of the plasma membrane. In inflammasome-activated macrophages, acute IL-1β secretion is induced by the concurrent, caspase-1-mediated cleavage of gasdermin D (122). The N-terminal domain of gasdermin D forms a 215 Å pore of 31- to 34-fold symmetry on the plasma membrane (Fig. 2*C*) (123, 124). It was hypothesized that IL-1β is rapidly released through the large pore, and the negatively charged inner surface of the pore acts as an “electrostatic filter” to prevent negatively charged proteins (*e.g.*, pro-IL-1β) from passing through the pore (123).

A key challenge associated with any pore/channel mechanism is substrate specificity, that is, how does a lipidic or proteinaceous pore allow structurally diverse cognate substrates (*e.g.*, FGF2 and HIV-Tat) to pass through but not other cellular contents such as small ions and nonsubstrate proteins? Nor can it explain how a 70 kDa fusion protein consisting of FGF2, GFP, and dihydrofolate reductase (DHFR) (FGF2–GFP–DHFR) is secreted with nearly the same efficiency as the 18 kDa FGF2 (47), as one would expect the attachment of a large, folded cargo domain to sterically interfere with membrane insertion, pore formation, and/or movement through the pore. Further, the pore model does not reconcile the following observations on IL-1β release: (1) the polybasic motif (^88^KNYPKKK^94^) is required for both gasdermin D–independent and gasdermin D–dependent release of IL-1β (122) or (2) the fast opening and closing of the putative pore in a PI(4,5)P2-dependent fashion (125).

#### Peroxisomal membrane pores

A transient, highly dynamic pore of up to 90 Å in size has been proposed to transport folded proteins across the peroxisomal membrane, largely based on results from electrophysiological studies (126). This hypothesis is supported by the observation that yeast Pex5 and Pex14 proteins, which are essential for protein import into the peroxisome *in vivo,* can form an ion-conducting channel *in vitro* (127). However, it is unclear how the channel, whose structure is currently unknown, adapts to transporting different-sized cargo molecules (with diameters of up to 126 Å) while maintaining the membrane barrier function. The channel mechanism also cannot explain how the PTS receptors (Pex5 or Pex7) are recycled back to the cytosol in a step requiring ubiquitination and ATP hydrolysis (128).

#### Gated mitochondrial membrane pore

The folded Rip1 protein is translocated from the mitochondrial matrix into the inner membrane by the Bcs1 AAA protein (112). How it might do so was recently illuminated from the cryo-EM structures of the *Saccharomyces cerevisiae* and mouse Bcs1 AAA proteins (129). Structurally, Bcs1 has a distal AAA and proximal middle domain in the matrix and a transmembrane (TM) domain spanning the inner membrane. Bcs1 forms a homoheptameric structure and has two large vestibules (both large enough to accommodate the folded Rip1), one located in the matrix and one located in the inner membrane (Fig. 2*D*). In the apo structure of the *S. cerevisiae* Bcsa AAA complex, the entrance to the matrix vestibule is smaller compared to that when ADP is bound (27 Å *versus* 40 Å). Conversely, Tang *et al*. (130) showed with the mouse Bcs1 AAA protein that the entrance is dramatically smaller in the [γ-S]ATP state compared to the apo and ADP state (20 Å *versus* 40 Å). Both studies reveal dramatic nucleotide-dependent conformational changes between the matrix vestibule and the inner membrane vestibule.

Kater *et al*. (129) proposed an airlock-like mechanism to account for the translocation of the folded Rip1 by Bcs1 AAA (Fig. 2*D*). Step 1 is the loading step, in which the substrate can access the matrix vestibule *via* the wide opening entrance, but it cannot access the inner membrane vestibule since the gate is mostly closed. In step 2, the gating step, the seal-forming middle domain between the two vestibules opens, allowing the protein to move into the inner membrane vestibule. As the gate between the two vestibules opens, the outer matrix vestibule gate facing the matrix closes. In Step 3, the release step, the hydrophilic Fe-S domain of Rip1 is translocated across the inner membrane vestibule to the intramitochondrial space and the N-terminal TM segment is laterally integrated into the inner membrane. This proposal is supported by the structure of [γ-S]ATP-bound form (130). In the latter structure, there is a dramatic constriction of the matrix vestibule such that it cannot accommodate a folded structure. Further structural studies are needed to shed light on how the inner membrane domain can open on the intramembrane side to allow the folded cargo to transfer to the intramitochondrial space.

### Local membrane disruption

Some proteins enter the cell directly by translocating across the plasma membrane. An example is the adenylate cyclase toxin (CyaA) secreted by *Bordetella pertussis*, the causative agent for whooping cough (131). CyaA contains an N-terminal adenylate cyclase (AC) domain followed by a C-terminal hydrophobic hemolysin domain, which is responsible for translocating the AC domain across the plasma membrane. AC translocation has been reconstituted *in vitro* using an artificial lipid bilayer (designed to mimic the plasma membrane), requiring only the presence of Ca^2+^ ions and a negative membrane potential but no additional host factors (132). It has been proposed that an α-helical peptide located within the hemolysin domain locally disrupts membrane bilayer integrity to allow the AC domain to cross the membrane (133, 134). Local destabilization and/or disruption of the endosomal membrane has also been invoked to explain the endosomal escape of nonenveloped viruses (135) and other biological cargos, such as nucleic acids delivered by cationic polymers (136). In addition, membrane destabilization has been proposed to mediate the transport of folded proteins across the plasma membrane in the opposite direction, for example, during the unconventional secretion of FGF1 (48). However, this model faces the same difficulties that have been described for the pore/channel mechanism. It is currently unknown how the proteins disrupt the cell membrane, how the disrupted membrane structure allows the “intended” proteins to cross (but not other molecules), or how the disrupted membrane is subsequently repaired.

### Protein-induced membrane thinning

A new paradigm in the protein transport field is the translocation of a protein across a distorted or thinned membrane (137). Membrane distortion and thinning may be caused by the presence of short TM segments and/or the formation of a hydrophilic groove open to the lipid bilayer and the aqueous compartment outside the membrane. This model has been invoked to rationalize the transport of folded proteins by the TAT system and the ER-to-cytosol translocation of nonenveloped viruses as largely intact particles (111). For the poliovirus SV40, there are structural changes that are triggered by protein disulfide isomerase that expose the hydrophobic surfaces of VP2 and VP3 (107, 108). The hydrophobic virus binds and inserts into the ER membrane after being released by BiP and other chaperones. For the insertion across the membrane, the ER membrane protein EMC1, a key component of the multisubunit ER membrane protein complex is required, which is known to thin the membrane (110). In addition, the cytosolic extraction machinery (Hsc70-Hsp105-SGTA-Bag2) is required to eject the viral particle from the ER membrane to the cytosol (138, 139, 140, 141).

**Figure 3:**
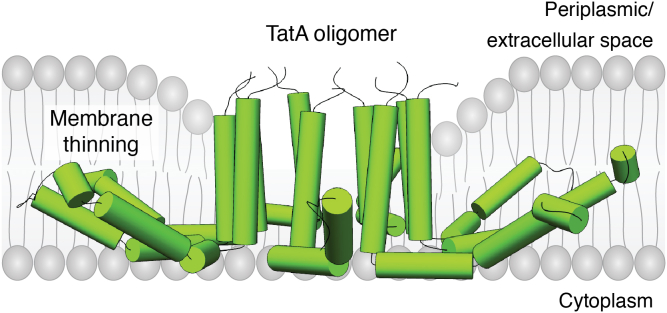
**Membrane-thinning mechanism.** A model of a TatA oligomer (9-mer) in a detergent micelle (PDB 2LZS) (145) is positioned in a phospholipid bilayer. The hydrophobic mismatch between the short TatA TM segment and membrane causes thinning of the bilayer around the TatA oligomer. This figure was adapted from Figure 3*D* in (7). PDB, Protein Data Bank; TM, transmembrane.

The *E. coli* TAT system consists of three integral membrane proteins TatA, TatB, and TatC (20, 22, 142, 143). TatA and TatB each contain a single TM helix and a cytoplasmic amphipathic helix, with the N terminus facing the periplasm. TatA forms oligomeric rings of different sizes (144). NMR studies of the TatA oligomer in detergent show that Gln8 located in the TM helix is in an aqueous environment and points toward the center of the oligomer, resulting in a short hydrophobic pore in the center of the complex (145). The amphipathic helix of TatA extends outward permitting the formation of different sized oligomers. TatB and TatC also form an oligomeric complex (TatBC) (146) and function as the receptors for substrates (147, 148, 149). Upon substrate binding to TatBC through their twin-arginine signal sequence, multiple TatA oligomers are recruited to the TatBC–substrate complex to form a large multimeric assembly as the functional translocase (148, 150, 151, 152). The TM helix of TatA is very short, and it is believed that hydrophobic mismatch between the membrane bilayer and the oligomeric TM helices cause the membrane to thin (Fig. 3). Molecular dynamics simulation studies of a 4- and 9-mer oligomer revealed dramatic membrane thinning in an *E. coli* lipid membrane. The hydrophobic lipid phase decreases to about half the normal size of a regular membrane. The TM helices are perpendicular to the membrane while the amphipathic helices are parallel to the membrane (145). The TatABC–substrate complex is subsequently believed to be translocated through the destabilized membrane, possibly by a pulling force. After translocation is complete, the twin-arginine signal peptide is cleaved by a signal peptidase and the TatABC complex is disassembled.

It was hypothesized that TatA oligomers of varied sizes form different sized hydrophobic pores that enable different-sized substrates to be translocated by the TAT system. In the center of the pore, the lipids are distorted compared to bulk lipids (145) and must move to the side during the transport of a folded protein. The substrate protein is surrounded by lipid molecules as it is being translocated across the pore; this may help seal the membrane, although some ion leakage is expected. The phage shock protein A (pspA) and its homolog in chloroplasts, both of which are implicated in the maintenance of membrane integrity, have been proposed to minimize the leakage of ions during Tat-dependent transport (153, 154). This model is supported by the observations that PspA binds to *E. coli* TatA protein and that the *Bacillus subtilis* homolog LiaH copurifies with the TatAyCy complex (155, 156). Additional evidence that TatA destabilizes the *E. coli* membrane upon substrate binding came from the studies by Bruser *et al.* (157), who showed that the TM helix of TatA causes destabilization of the membrane due to its short length. However, this destabilizing effect of the TM segment is normally compensated for by the amphipathic helix that has its amino-terminal half embedded into the membrane. They showed that the addition of substrate causes a reorientation of the amphipathic helix, which leads to a weakening of the membrane. A major limitation of the membrane-thinning hypothesis is that it cannot explain how proteins without any TM segment (*e.g.*, NleC, FGF2, and IL-1β) move across cellular membranes. It also has difficulty in explaining why (or how) the TatABC complex translocates up to eight substrate proteins simultaneously (vide infra).

### Transport by membrane vesicles

**Figure 4:**
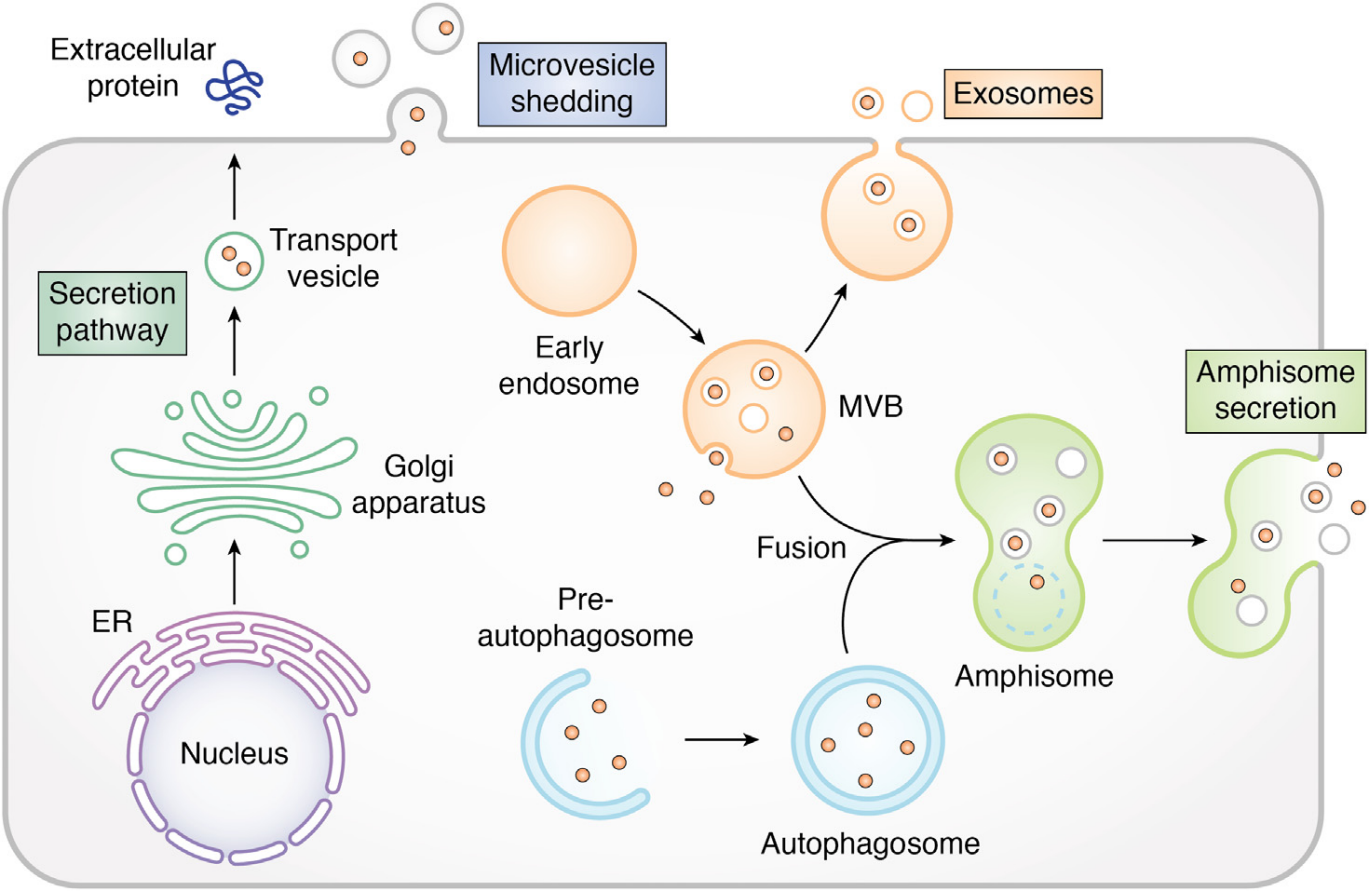
**Membrane transport of folded proteins by vesicular structures.** The conventional secretion pathway (ER–Golgi –transport vesicle–plasma membrane) is shown on the left, while the unconventional secretion pathways (microvesicle shedding, exosome, and amphisome secretion) are on the right. ER, endoplasmic reticulum.

Instead of physically passing through a lipid bilayer, proteins may be packaged into membrane-bound vesicles or structures and transported across a cell membrane. Three different vesicular transport mechanisms have been proposed: (1) secretion by microvesicles pinching off from the plasma membrane, (2) entering the endolysosomal system through incorporation into multivesicular bodies (MVBs) or lysosomes, and (3) engulfment by autophagosomes or autophagy derived structures such as amphisomes (Fig. 4). The latter two mechanisms constitute the type III UPS pathway (45, 46). After crossing the membrane barrier, the protein may be released into the solution or remain enveloped inside the vesicular structure.

#### Microvesicle formation

Microvesicles are shed directly from the plasma membrane by pinching the plasma membrane outward in a process like viral budding (Fig. 4). Cytosolic and membrane proteins are recruited into microvesicles as they form. Microvesicles differ from exosomes by having larger sizes (typically 50–1000 nm *versus* 30–150 nm in diameter) and different protein contents. The shedding of microvesicles from the plasma membrane is partially responsible for the UPS of IL-1β in P2X7 receptor–stimulated lipopolysaccharide-treated THP-1 cells (158) and thrombin-activated platelets (159). In THP-1 cells shedding of IL-1β-containing microvesicles is preceded by flip of phosphatidylserine to the outer leaflet of the plasma membrane (158). The IL-1β contained in shed microvesicles is bioactive and may be released following contact with IL-1 receptor (IL-1RI) expressing cells (158). Stimulation of the microvesicles (which express P2X7R) with extracellular ATP induces lysis of the microvesicles and the release of their contents into the extracellular environment (160). This provides a mechanism for release of the protected IL-1β at target sites to elicit cellular responses. Other examples of proteins secreted by this mechanism include Fas-associated protein with death domain (161) and focal adhesion kinase (162). Enveloped viruses adopt the reverse process, membrane fusion with the plasma or endosomal membrane, to transfer their proteins and genetic materials into eukaryotic cells (163). Researchers have also encapsulated protein and nucleic acid cargos into membrane vesicles and delivered them into the cell (164).

#### Endosomal secretion

Cytosolic proteins can also be secreted in a protected form by being packaged and secreted *via* exosomes (165). Exosomes are small vesicles that are secreted from MVBs (or late endosomes). They are formed by the inward budding of the endosomal membrane and contain the cytosol proteins to be secreted (Fig. 4). The resulting intraluminal vesicles (which contain the cargo protein) have two fates. They can be unconventionally secreted as exosomes upon fusion of the MVB with the plasma membrane (166, 167). Alternatively, proteins in intraluminal vesicles can be degraded upon fusion of the MVB with the lysosomes. Several unconventionally secreted proteins, including IL-1β (168) and enolase (165), have been reported to utilize exosomes as one of the secretory mechanisms (type III UPS). Note that proteins secreted by this mechanism remain enveloped inside the exosome and are not immediately available for function in the extracellular environment. It has been hypothesized that the membrane coating may protect IL-1β from degradation, increasing its lifetime in circulation and allowing it to travel to and initiate signaling processes at sites distant to the local inflammatory lesion (169). But how the cargo proteins are released from the exosomes to function in the extracellular environment is currently unknown. Recently, Zhang *et al*. showed that some leaderless proteins (*e.g.*, IL-1β) are translocated into the ER–Golgi intermediate compartment by the integral membrane protein TMED10 and subsequently transported *via* small vesicles to the plasma membrane (170). Fusion of the vesicles with the plasma membrane releases the leaderless proteins in their free form into the extracellular environment.

#### Amphisome secretion

Eukaryotic cells can leverage the autophagy pathway to secret some proteins unconventionally (Fig. 4). During classical autophagy, damaged proteins or organelles in the cytosol are enveloped into a double-membrane structure. The resulting vesicle (or autophagosome) fuses with the lysosome to form an autolysosome resulting in the proteolytic degradation of its contents (171). However, autophagosomes sometimes fuse with MVBs to form structures called amphisomes (172). Amphisomes then fuse with the plasma membrane and deliver cargo to the external environment as a type III mechanism of UPS, or fuse with the lysosome, where their contents are degraded. The specific molecular signal that causes amphisomes to fuse with the plasma membrane rather than the lysosome remains unknown.

Histone H3 is one of the best characterized proteins that are unconventionally secreted by this mechanism (165). Histone H3 is taken up into an LC3 (an autophagy marker)-positive autophagosome. Next, the autophagosome matures and its inner membrane is degraded. The autophagosome fuses with CD63 (an MVB marker)-positive endosomes to form an amphisome. Finally, the amphisome fuses with the plasma membrane and releases H3 in a nonvesicular form. Acyl-CoA–binding protein (AcbA in *Dictyostelium*, Acb1 in yeast, and ACBP in mammalian cells) is another well-studied cargo protein using the type III UPS pathway. Malhotra *et al.* used *Dictyostelium* as a model system to study the role of GRASP protein (GrpA in *Dictyostelium* and Grh1 in yeast) and found that a GRASP KO strain failed to form viable spores (40). They showed that grpA^-^ cells cannot secrete AcbA, which is required for spore formation. Similarly, upon starvation, yeast secretes Acb1 (the yeast ortholog of AcbA) *via* a cup-shaped (termed as CUPS) compartment in a Grh1-dependent manner (39). This CUPS structure represents a subpopulation of autophagosomes whose formation depends on PI(3)P, ESCRT-I, II, III components but is independent of AAA-ATPase Vps4 function (31, 38, 39). ESCRT-III component Snf7 is recruited to CUPS compartment and the plasma membrane t-SNARE Sso1 is required for the subsequence membrane fusion of CUPS and release of Acb1 protein (37, 39). These studies provide a paradigm of how the unconventional secretion of Acb1 is related to the CUPS compartment, which depends on autophagosome, ESCRTs, and Grh1.

## Translocation of folded proteins by VBC

**Figure 5:**
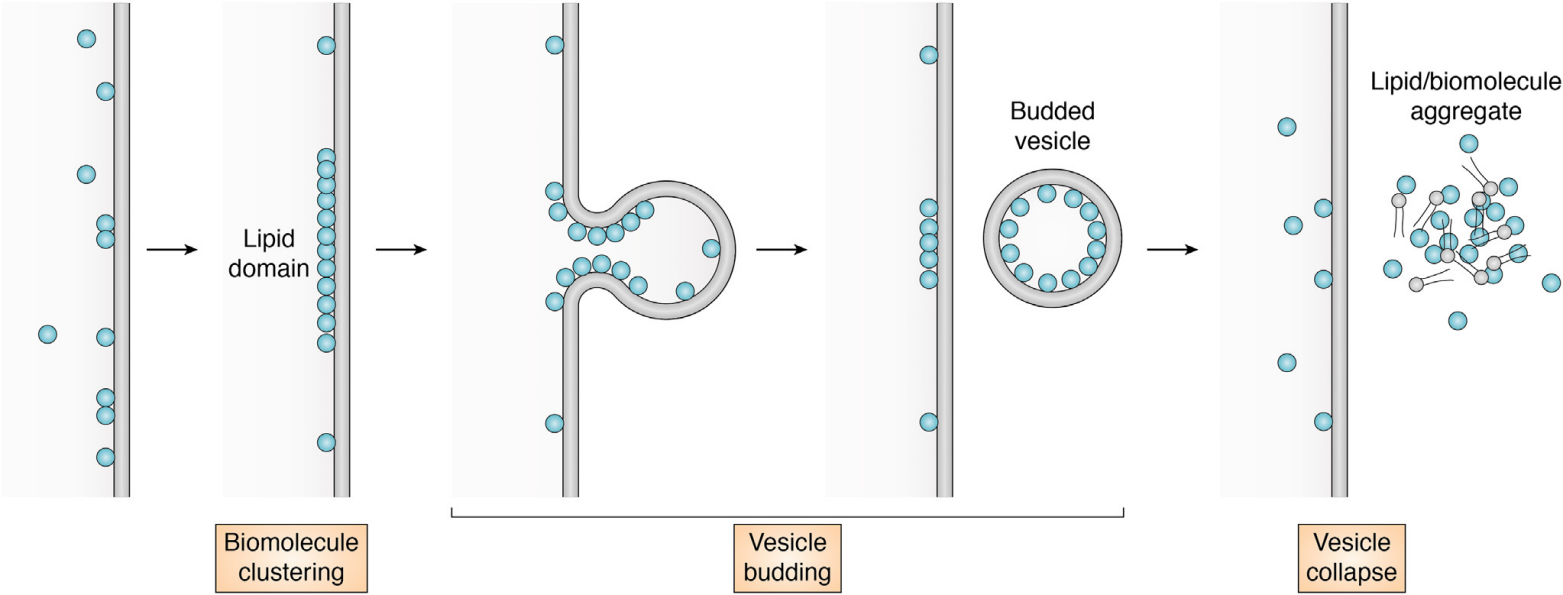
**Membrane translocation by the VBC mechanism.** Biomolecules (indicated by the *cyan spheres*) first bind to the *cis* side of a membrane and are evenly distributed over the membrane. They then cluster together to induce the formation of a lipid domain, which buds off as a small vesicle. Collapse of the vesicle releases the biomolecules (and phospholipids) to the *trans* side of the membrane. VBC, vesicle budding and collapse.

The VBC mechanism was first discovered during our investigation of the endosomal escape of CPPs (173, 174). We subsequently showed that bacterial toxins DT and NleC also escape the endosome by the VBC mechanism (13). A survey of the literature led us to hypothesize that VBC may be a novel, fundamental membrane transport mechanism that drives the translocation of a variety of biomolecules/systems including peptides, folded proteins, nonenveloped viruses, and various synthetic drug delivery vehicles (*e.g.*, polyplexes, lipoplexes, and lipid nanoparticles) across different cellular membranes (175). During VBC, the biomolecules bind to the phospholipids of a membrane and cluster the phospholipids into a lipid domain(s) (Fig. 5). The formation of the lipid domain generates line tension between the lipid domain and the surrounding membrane, which causes the lipid domain to bud out as a small vesicle (176). The budded vesicle then spontaneously and rapidly collapses, presumably because of the inherently unstable nature of the small vesicle. In some cases, the vesicle collapses as it buds off the cell membrane (173). The disintegrated vesicle initially forms an amorphous aggregate consisting of both membrane lipids and biomolecules, which slowly dissolves into the bulk solution on the other side of the membrane.

### What structural features facilitate VBC?

To promote VBC, the biomolecule must be able to bind to the cell membrane and induce the clustering of biomolecule-bound phospholipids into lipid domains. Immediately before the budded vesicle pinches off, the budding neck requires distortion of the membrane structure from the lamellar shape into the “saddle-splay” shape, which features negative Gaussian curvature (*i.e.*, simultaneous positive and negative curvatures in orthogonal directions) and has higher potential energy than the “ground states” present before or after the budding event. To “catalyze” the budding event, the biomolecules must bind selectively to the budding neck and reduce the energy barrier of the VBC event. To do so, the biomolecules need to induce positive and negative membrane curvatures simultaneously. A key observation was that biomolecules highly effective in inducing VBC are typically amphipathic and conformationally constrained (177), as exemplified by cyclic CPP12 (cyclo(Phe-D-Phe-Nal-Arg-D-Arg-Arg-D-Arg-Gln), where Nal is L-naphthylalanine) (173). Conformational rigidity increases the membrane-binding affinity of a biomolecule, while amphipathicity facilitates the formation of negative Gaussian curvature at the budding neck. Insertion of hydrophobic groups (*e.g.*, the side chains of Nal and Phe) in between phospholipid molecules generates positive membrane curvature, while polybasic groups (*e.g.*, arginine residues and, less effectively, lysine residues) induce negative curvature by hydrogen bonding to and bringing together the phosphate head groups of phospholipids (177). Time-lapse confocal microscopic experiments confirmed that CPPs (173, 174) and bacterial protein toxins (13) are indeed concentrated at the budding neck during VBC. A linear correlation between the endosomal escape efficiency and the endosomal membrane-binding affinity was observed for a panel of structurally diverse CPPs (173).

### VBC is uniquely suited for the membrane transport of folded proteins

Compared with other membrane translocation mechanisms, the VBC mechanism has several unique features. First, the biomolecule/system crosses the membrane topologically (*i.e.*, from one side to the other side of the membrane) but not physically (*i.e.*, without going through the lipid bilayer). This feature renders VBC compatible with biomolecules of any size or physicochemical property, so long as they contain structural elements that interact with and induce negative Gaussian curvature on the target membrane. The VBC mechanism thus readily explains how FGF2 (18 kDa) and FGF2–GFP–DHFR (70 kDa) were unconventionally secreted with similar efficiencies (47) or how 90 Å gold nanoparticles and 126 Å oligomeric proteins are imported into the peroxisome (178). Second, membrane translocation by VBC does not require unfolding of the protein and therefore explains how cofactor-containing enzymes and noncovalent protein complexes are transported across the plasma (*e.g.*, export of Ni-Fe hydrogenase (179) by the TAT system), endosomal (*e.g.*, the delivery of noncovalently associated nucleic acids by DT (82, 83)), or the peroxisomal membrane (*e.g.*, the “piggyback” transport of proteins without PTS1/PTS2 into the peroxisome (57, 178, 180, 181). The conformational rigidity of folded proteins may serve as a potential quality control mechanism during their translocation by the TAT and UPS pathways—folded proteins bind effectively to the budding neck and are translocated, whereas unfolded proteins do (are) not. However, it should be stressed that a folded structure is not a prerequisite for VBC, as linear CPPs and nonpeptidic molecules also cross the endosomal membrane by VBC (173, 174, 175). Third, the cell membrane remains intact before, during, and after each VBC event. The VBC mechanism therefore reconciles one of the most perplexing observations—that different-sized proteins, protein complexes, and nanoparticles are transported across a cell membrane without compromising its barrier function. In contrast, any mechanism that involves a protein physically traversing a cell membrane would require partial or total disruption of the membrane. Note that a limited transfer of ions and other contents is expected during VBC, as each VBC event results in the release of a small volume of the donor compartment into the recipient compartment. Finally, the VBC mechanism is energy independent, although it may be facilitated by the presence of membrane potentials and/or transmembrane pH gradients (*e.g.*, across the endosomal membrane). This makes VBC highly versatile and potentially operative in any cellular compartment.

### Bacterial toxins escape the endosome by VBC

We recently demonstrated that bacterial toxins DT and NleC escape the endosome by inducing VBC (13). Binding of the R-domain of DT to its receptor on the host cell surface, the heparin-binding EGF-like growth factor (HB-EGF) receptor, results in the endocytosis of the receptor–DT complex. Endosomal acidification induces a conformational change of the T-domain, which inserts into the endosomal membrane to form an ion-conducting pore/channel. Instead of the T-domain acting as a pore to translocate the unfolded A-domain as previously proposed (77), we hypothesize that membrane insertion of the T-domain serves to increase the binding affinity of DT for the endosomal membrane, so that a minimum number of DT molecules can be concentrated into a single endosome to induce VBC (13). We estimated that a minimum of 80 to 360 biomolecules is required for each VBC event (175), corresponding to an endosomal concentration of 2 to 9 μM, which is much higher than physiological DT concentrations in the extracellular environment (pM to nM). Additional interactions between the endosomal membrane and other elements of DT, including amphipathic helices 1 and 2 of the T-domain (which contain both positively charged and hydrophobic residues), probably cause the DT-bound lipids to cluster into a toxin-enriched lipid domain. VBC from the lipid domain results in the simultaneous release of multiple DT molecules into the cytosol. At high DT concentrations, multiple VBC events may occur on the same endosome, either simultaneously or sequentially, until the vesicle is mostly depleted of the cargo.

DT- and NleC-mediated VBC events have been observed in real-time in HeLa (human cervical cancer) cells by live-cell confocal microscopy (13). Briefly, HeLa cells were simultaneously treated with a fluorescently (green) labeled phosphatidylserine and pHAb-labeled DT (or NleC). pHAb is a pH-sensitive dye (p*K*a = 6.0), which fluoresces (red) in the acidic endosomes/lysosomes (pH 4.5–6.5) but not in the extracellular or cytosolic environment (pH 7.4). This resulted in the selective and dual labeling (both green and red) of the endosomes/lysosomes, allowing direct visualization of vesicle budding from the endosomal membrane. Collapse of the budded vesicles was indicated by the sudden loss of the red (but not the green) fluorescence from the budded vesicle (or its remnant). Further, the endosomes were enlarged by the treatment with a kinase inhibitor, allowing the VBC intermediates to be captured by confocal microscopy (13). Additional support for the VBC mechanism came from an earlier observation that the endosomal release of DT follows a “quantal” kinetics: ∼80 DT molecules are simultaneously released from an endosome (*i.e.*, as a “bolus”), irrespective of the extracellular DT concentration (182).

### Potential involvement of VBC in the UPS system

The experimental evidence used to support the pore model for the type I UPS pathway are also consistent with the VBC mechanism. For example, FGF2, HIV-Tat, and IL-1β (which all undergo type I UPS) share the common properties of membrane binding and PI(4,5)P2-dependent oligomerization. These properties also facilitate phospholipid clustering and the formation of lipid domains during VBC. Protein oligomerization is likely the consequence of their binding to and clustering of phospholipids, as observed for CPPs and bacterial toxins (13, 174, 175). The transport of small molecules (*e.g.*, Alexa488 dye) across the GUV membrane through PI(4,5)P2-dependent “pores” (33) can be alternatively explained by VBC events. A key difference between the two mechanisms is the kinetics of protein translocation—translocation through a pore is sequential, whereas a “bolus” of biomolecules is simultaneously transported by each VBC event. Dimou *et al*. recently employed high-resolution total internal reflection fluorescence microscopy to visualize single events of FGF2-GFP recruitment at the inner leaflet and FGF2-GFP translocation to the outer leaflet of the plasma membrane in living cells (183). Oligomerization of FGF2-GFP on the inner leaflet was found to be a relatively “slow” process, whereas translocation of the oligomers to the outer leaflet occurred instantaneously (in <200 ms), suggesting that the FGF2-GFP oligomers were transported across the plasma membrane as a “bolus.”

In the case of IL-1β, cleavage by caspase-1 increases its pI from 4.6 to 8.8 and exposes a polybasic motif for membrane binding and possibly inducing VBC (122). The gasdermin D–independent IL-1β release is slow, presumably because the polybasic motif (^88^KNYPKKK^94^) is not optimal for inducing VBC (*e.g.*, the lack of arginine residues). In the presence of gasdermin D N-terminal domain, which contains several arginine-rich motifs, the IL-1β–gasdermin D complex may induce more robust VBC. Indeed, the expression of gasdermin D N-terminal domain in HeLa cells resulted in robust budding and collapse of large vesicles from the plasma membrane, which was previously described as membrane “swelling” and “rupture” (124). The VBC mechanism explains why the polybasic motif of IL-1β is necessary for both gasdermin D–independent and gasdermin D–dependent IL-1β release (122). It offers a possible explanation for the gasdermin D–induced “calcium flares” inside the cells, usually near the plasma membrane (*i.e.*, VBC in the inward direction), which were previously interpreted as calcium influx following the opening of a 215 Å gasdermin D pore (125). It reconciles the phosphoinositide-dependent “pore dynamics” and the superiority of PI(3,4,5)P3, which should further enhance the binding affinity of IL-1β and gasdermin D for the plasma membrane, for inducing the gasdermin D activity (125). Finally, it provides a potential avenue for gasdermin D to selectively release certain proteins (*e.g.*, IL-1β) but not others (*e.g.*, pro-IL-1β and lactate dehydrogenase).

The type III UPS pathway and microvesicle shedding have been reported to release cytosolic proteins into the extracellular environment in the nonvesicular form (8). Note that the collapse of exosomes and microvesicles after their release from the cell represents a variation of the VBC mechanism. Interestingly, a new type of nonvesicular nanoparticles secreted by eukaryotic cells, termed “exomeres,” has recently been discovered (184). Exomeres (typically <50 nm in diameter) are enriched in extracellular matrix proteins, components of the proteasome, metabolic proteins (*e.g.*, hexokinase, glucose-6-phosphate isomerase, GAPDH, pyruvate kinase, and enolase), and nucleotide-binding proteins (*e.g.*, Argonaut and APP) and contain trace amounts of lipids that are common to microvesicles. It is tempting to suggest that these exomeres may come from collapsed microvesicles (and/or exosomes).

### Potential involvement of VBC in the TAT system

To our knowledge, the VBC mechanism is consistent with the literature on the TAT system. TatA contains an amphipathic, polybasic α-helix, which lies parallel to the inner leaflet of the plasma membrane and interacts with the membrane (145, 185). The α-helix therefore possesses the requisite structural elements and is properly oriented for binding to the plasma membrane and potentially inducing negative Gaussian curvature on the membrane. Indeed, the insertion of TatA alone into the lipid bilayer of large unilamellar vesicles (LUVs) caused “quantized,” partial, and temporary leakage of calcein (a fluorescent dye) from the vesicles (186). Translocation of substrate proteins requires the oligomerization of TatA (187). In the resting state, TatA is evenly distributed on the plasma membrane; upon substrate binding to TatBC, TatA protomers cluster around the TatBC receptor complex to form a large, multimeric complex as the functional translocase, which is readily visible by fluorescence microscopy (188). In thylakoid membranes, the fully assembled TAT translocase is a 2.2 MDa complex consisting of 208 Tha4 (TatA), eight Hcf106 (TatB), and eight cpTatC protomers and capable of transporting up to eight protein substrates at a time (187). This phenomenon is consistent with the formation of a protein-bound lipid domain, which is a prerequisite for VBC. The number of TatA protomers in a functional translocase (208) agrees with our previous estimate that each VBC event requires a minimum of 80 to 360 curvature-inducing molecules (175).

The VBC mechanism reconciles previous observations that cannot be explained by any of the competing models (*e.g.*, the membrane pore and thinning models). For example, the VBC mechanism does not cause any loss of membrane integrity and is compatible with protein substrates of any size, oligomeric state, or folding status. It thus explains how the TAT system exports protein substrates of varied sizes without causing significant membrane leakage (189, 190). On the other hand, each VBC event results in the transfer of a small volume from the *cis* to the *trans* side of the membrane, therefore explaining the “quantized,” temporary release of calcein from TatA-treated LUVs (186). Calcein release through a pore in the LUV membrane would be continuous, until an equilibrium is reached between the two sides of the membrane. The VBC mechanism allows multiple cargo molecules to be transported as a “bolus” and is consistent with the observation that the TAT translocase is capable of transporting protein oligomers (either noncovalent or covalent) by engaging multiple TatBC receptors simultaneously (191, 192). The TAT system has been shown to transport protein substrates that are covalently attached to the TatBC receptor complex (193). This is fully consistent with the VBC mechanism, during which the TatABC complex and the substrate move together as a unit, but more difficult to explain by a pore or membrane thinning mechanism, during which the substrate must shift in position relative to TatABC and covalent crosslinking would be expected to interfere with this relative movement. After the translocation of substrate proteins, some of the TatABC components may need to be recycled back to the cytosol. Since TatA alone apparently induces VBC *in vitro* (186), it is conceivable for the exported TatA to recycle back into the cytoplasm through an inward VBC event. A potential difficulty with the VBC mechanism is that the narrow periplasmic space of Gram-negative bacteria (∼20 nm between the outer and inner membranes in *E. coli*) may not be able to accommodate the budded vesicles, which have diameters of 50 to 100 nm (174). However, local separation of the outer and plasma membranes of bacterial cells by as much as 100 nm has been observed by electron microscopy (194). Moreover, the locally expanded periplasmic regions contained large vesicle-like objects (194).

### Potential involvement of VBC during the cellular entry of other proteins

The cell-permeable proteins previously discussed usually contain amphipathic or polybasic sequence motifs that can induce negative Gaussian curvature and VBC. For example, histone proteins are highly basic because of their need to bind to nucleic acids. PTEN-long contains an N-terminal hexaarginine motif that mimics CPPs (*e.g.*, R9) and was shown to be critical for the cellular entry of PTEN-long (94). The RRRRWWW motif used to engineer cell-permeable proteins is an efficient CPP in isolation (98). Cell-permeable antibodies (*e.g.*, TMab4) contain a hydrophobic motif, WYW (or similar sequences), in the CDR3 loop and polybasic sequences in the CDR1 and CDR2 loops of their VL domain (99). Adenovirus protein VI, which is responsible for the endosomal escape of the virus, contains a 20 aa amphipathic α-helix at its N terminus (85). This peptide binds to GUVs mimicking the endosomal membrane with an apparent *K*_D_ value of 3 μM, induces membrane curvature, and causes the GUVs to fragment into smaller vesicles or form tubular structures and peptide/lipid aggregates (85). These properties are reminiscent of those of cyclic CPPs, which exit the endosome by VBC (174). The phospholipase A type 2 domain of parvovirus protein VP1 mediates the endosomal escape of parvoviruses (87). It likely promotes VBC by producing lipid molecules that stabilize the negative Gaussian curvature at the budding neck. PLA2 hydrolyzes phosphatidylcholine into lysophosphatidylcholine and fatty acids. While phosphatidylcholine has an intrinsic lipid curvature of ∼0, lysophosphatidylcholine and fatty acids generate positive and negative membrane curvatures, respectively. Further experimentation will be necessary to ascertain whether these proteins/viruses escape the endosome by VBC, for example, by labeling them with pHAb and monitoring their intracellular trafficking by time-lapse confocal microscopy.

## Conclusion and future directions

It is now clear that membrane translocation of folded proteins is an integral component of cellular biogenesis and function in all three domains of life. In addition to the previously established systems (TAT, UPS, and protein import into the peroxisome), we survey the evidence that folded proteins are also transported through the endosomal membrane and possibly the ER membrane of eukaryotic cells as well as the outer membrane of Gram-negative bacteria. Protein transport in the folded state provides an important alternative to the conventional pathways such as the Sec system, which is ineffective for transporting proteins containing complex metal cofactors or noncovalent protein complexes. Compared with the Sec pathway, the TAT and UPS systems allow functional proteins to be transported across a cellular membrane, without competing for the same translocon (*e.g.*, SecYEG) with many other proteins. However, this alternative pathway is likely limited to only a subset of proteins, presumably those that contain proper amphipathic/polybasic sequences (or surfaces) and their associated cargo proteins. The molecular mechanism by which folded proteins translocate across cell membranes remains incompletely understood. Our analysis of the literature data suggests that some of the systems discussed in this review (including TAT, UPS, and cellular entry of proteins) are potentially mediated by the VBC mechanism. However, additional research is warranted to validate or disprove this hypothesis as well as the alternative mechanisms previously proposed by others (*e.g.*, pore/channel formation and membrane disruption/thinning). Additionally, many molecular details of the VBC mechanism, for example, how proteins (and other biomolecules) interact with a lipid bilayer to induce negative Gaussian curvature during VBC and why or how the budded vesicle collapses, are currently unresolved. Finally, the discovery of VBC as a novel mechanism for the membrane translocation of CPPs and bacterial toxins opens a door to the rational design of cell-permeable peptides, proteins, and other drug delivery vehicles.

## Conflict of interest

The authors declare that they have no conflicts of interest with the contents of this article.

## References

[1] Chen Y., Zhang Y., Yin Y., Gao G., Li S., Jiang Y. (2005). SPD - a web-based secreted protein database. Nucl. Acids Res..

[2] Teufel F., Almagro Armenteros J.J., Johansen A.R., Gislason M.H., Pihl S.I., Tsirigos K.D. (2022). SignalP 6.0 predicts all five types of signal peptides using protein language models. Nat. Biotechnol..

[3] Wickner W., Schekman R. (2005). Protein translocation across biological membranes. Science.

[4] Schatz G., Dobberstein B. (1996). Common principles of protein translocation across membranes. Science.

[5] Rapoport T.A., Li L., Park E. (2017). Structural and mechanistic insights into protein translocation. Annu. Rev. Cell Dev. Biol..

[6] Zimmermann R., Eyrisch S., Ahmad M., Helms V. (2011). Protein translocation across the ER membrane. Biochim. Biophys. Acta.

[7] Cline K. (2015). Mechanistic aspects of folded protein transport by the twin arginine translocase (Tat). J. Biol. Chem..

[8] Dimou E., Nickel W. (2018). Unconventional mechanisms of eukaryotic protein secretion. Curr. Biol..

[9] Leon S., Goodman J.M., Subramani S. (2006). Uniqueness of the mechanism of protein import into the peroxisome matrix: transport of folded, co-factor-bound and oligomeric proteins by shuttling receptors. Biochim. Biophys. Acta.

[10] Williams J.M., Tsai B. (2016). Intracellular trafficking of bacterial toxins. Curr. Opin. Cell Biol..

[11] Auger A., Park M., Nitschke F., Minassian L.M., Beilhartz G.L., Minassian B.A. (2015). Efficient delivery of structurally diverse protein cargo into mammalian cells by a bacterial toxin. Mol. Pharm..

[12] Stolle A.S., Norkowski S., Korner B., Schmitz J., Luken L., Frankenberg M. (2017). T3SS-independent uptake of the short-trip toxin-related recombinant NleC effector of enteropathogenic *Escherichia coli* leads to NF-kappaB p65 cleavage. Front. Cell Infect. Microbiol..

[13] Sahni A., Pei D. (2021). Bacterial toxins escape the endosome by inducing vesicle budding and collapse. ACS Chem. Biol..

[14] Berks B.C. (2015). The twin-arginine protein translocation pathway. Annu. Rev. Biochem..

[15] Frain K.M., Robinson C., van Dijl J.M. (2019). Transport of folded proteins by the Tat system. Protein J..

[16] New C.P., Ma Q., Dabney-Smith C. (2018). Routing of thylakoid lumen proteins by the chloroplast twin arginine transport pathway. Photosynth Res..

[17] Mould R.M., Robinson C. (1991). A proton gradient is required for the transport of two lumenal oxygen-evolving proteins across the thylakoid membrane. J. Biol. Chem..

[18] Cline K., Ettinger W.F., Theg S.M. (1992). Protein-specific energy requirements for protein transport across or into thylakoid membranes. Two lumenal proteins are transported in the absence of ATP. J. Biol. Chem..

[19] Klosgen R.B., Brock I.W., Herrmann R.G., Robinson C. (1992). Proton gradient-driven import of the 16 kDa oxygen-evolving complex protein as the full precursor protein by isolated thylakoids. Plant Mol. Biol..

[20] Weiner J.H., Bilous P.T., Shaw G.M., Lubitz S.P., Frost L., Thomas G.H. (1998). A novel and ubiquitous system for membrane targeting and secretion of cofactor-containing proteins. Cell.

[21] Santini C.L., Ize B., Chanal A., Muller M., Giordano G., Wu L.F. (1998). A novel sec-independent periplasmic protein translocation pathway in *Escherichia coli*. EMBO J..

[22] Sargent F., Bogsch E.G., Stanley N.R., Wexler M., Robinson C., Berks B.C. (1998). Overlapping functions of components of a bacterial Sec-independent protein export pathway. EMBO J..

[23] Pohlschroder M., Gimenez M.I., Jarrell K.F. (2005). Protein transport in Archaea: sec and twin arginine translocation pathways. Curr. Opin. Microbiol..

[24] Petru M., Wideman J., Moore K., Alcock F., Palmer T., Dolezal P. (2018). Evolution of mitochondrial TAT translocases illustrates the loss of bacterial protein transport machines in mitochondria. BMC Biol..

[25] Berks B.C. (1996). A common export pathway for proteins binding complex redox cofactors?. Mol. Microbiol..

[26] Jongbloed J.D., van der Ploeg R., van Dijl J.M. (2006). Bifunctional TatA subunits in minimal Tat protein translocases. Trends Microbiol..

[27] Palmer T., Sargent F., Berks B.C. (2005). Export of complex cofactor-containing proteins by the bacterial Tat pathway. Trends Microbiol..

[28] Pallotta M.T., Nickel W. (2020). FGF2 and IL-1beta - explorers of unconventional secretory pathways at a glance. J. Cell Sci..

[29] Cohen M.J., Chirico W.J., Lipke P.N. (2020). Through the back door: unconventional protein secretion. Cell Surf..

[30] Kim J., Gee H.Y., Lee M.G. (2018). Unconventional protein secretion - new insights into the pathogenesis and therapeutic targets of human diseases. J. Cell Sci..

[31] Cruz-Garcia D., Curwin A.J., Popoff J.F., Bruns C., Duran J.M., Malhotra V. (2014). Remodeling of secretory compartments creates CUPS during nutrient starvation. J. Cell Biol..

[32] Schroder K., Tschopp J. (2010). The inflammasomes. Cell.

[33] Steringer J.P., Bleicken S., Andreas H., Zacherl S., Laussmann M., Temmerman K. (2012). Phosphatidylinositol 4,5-bisphosphate (PI(4,5)P2)-dependent oligomerization of fibroblast growth factor 2 (FGF2) triggers the formation of a lipidic membrane pore implicated in unconventional secretion. J. Biol. Chem..

[34] Graziani I., Bagala C., Duarte M., Soldi R., Kolev V., Tarantini F. (2006). Release of FGF1 and p40 synaptotagmin 1 correlates with their membrane destabilizing ability. Biochem. Biophys. Res. Commun..

[35] Popa S.J., Stewart S.E., Moreau K. (2018). Unconventional secretion of annexins and galectins. Semin. Cell Dev. Biol..

[36] Anjard C., Loomis W.F. (2005). Peptide signaling during terminal differentiation of Dictyostelium. Proc. Natl. Acad. Sci. U. S. A..

[37] Duran J.M., Anjard C., Stefan C., Loomis W.F., Malhotra V. (2010). Unconventional secretion of Acb1 is mediated by autophagosomes. J. Cell Biol..

[38] Curwin A.J., Brouwers N., Alonso Y.A.M., Teis D., Turacchio G., Parashuraman S. (2016). ESCRT-III drives the final stages of CUPS maturation for unconventional protein secretion. Elife.

[39] Bruns C., McCaffery J.M., Curwin A.J., Duran J.M., Malhotra V. (2011). Biogenesis of a novel compartment for autophagosome-mediated unconventional protein secretion. J. Cell Biol..

[40] Kinseth M.A., Anjard C., Fuller D., Guizzunti G., Loomis W.F., Malhotra V. (2007). The Golgi-associated protein GRASP is required for unconventional protein secretion during development. Cell.

[41] Rayne F., Debaisieux S., Bonhoure A., Beaumelle B. (2010). HIV-1 Tat is unconventionally secreted through the plasma membrane. Cell Biol. Int..

[42] Merezhko M., Brunello C.A., Yan X., Vihinen H., Jokitalo E., Uronen R.L. (2018). Secretion of Tau *via* an unconventional non-vesicular mechanism. Cell Rep.

[43] Chirico W.J. (2011). Protein release through nonlethal oncotic pores as an alternative nonclassical secretory pathway. BMC Cell Biol..

[44] Monteleone M., Stow J.L., Schroder K. (2015). Mechanisms of unconventional secretion of IL-1 family cytokines. Cytokine.

[45] Manjithaya R., Anjard C., Loomis W.F., Subramani S. (2010). Unconventional secretion of Pichia pastoris Acb1 is dependent on GRASP protein, peroxisomal functions, and autophagosome formation. J. Cell Biol..

[46] Ebner P., Gotz F. (2019). Bacterial excretion of cytoplasmic proteins (ECP): occurrence, mechanism, and function. Trends Microbiol..

[47] Backhaus R., Zehe C., Wegehingel S., Kehlenbach A., Schwappach B., Nickel W. (2004). Unconventional protein secretion: membrane translocation of FGF-2 does not require protein unfolding. J. Cell Sci..

[48] Prudovsky I., Kumar T.K., Sterling S., Neivandt D. (2013). Protein-phospholipid interactions in nonclassical protein secretion: problem and methods of study. Int. J. Mol. Sci..

[49] Kim P.K., Hettema E.H. (2015). Multiple pathways for protein transport to peroxisomes. J. Mol. Biol..

[50] Gould S.J., Keller G.A., Hosken N., Wilkinson J., Subramani S. (1989). A conserved tripeptide sorts proteins to peroxisomes. J. Cell Biol..

[51] Brocard C., Hartig A. (2006). Peroxisome targeting signal 1: Is it really a simple tripeptide?. Biochim. Biophys. Acta.

[52] Petriv O.I., Tang L., Titorenko V.I., Rachubinski R.A. (2004). A new definition for the consensus sequence of the peroxisome targeting signal type 2. J. Mol. Biol..

[53] Brul S., Westerveld A., Strijland A., Wanders R.J., Schram A.W., Heymans H.S. (1988). Genetic heterogeneity in the cerebrohepatorenal (Zellweger) syndrome and other inherited disorders with a generalized impairment of peroxisomal functions. A study using complementation analysis. J. Clin. Invest..

[54] Keller G.A., Gould S., Deluca M., Subramani S. (1987). Firefly luciferase is targeted to peroxisomes in mammalian cells. Proc. Natl. Acad. Sci. U. S. A..

[55] Walton P.A., Gould S.J., Rachubinski R.A., Subramani S., Feramisco J.R. (1992). Transport of microinjected alcohol oxidase from Pichia pastoris into vesicles in mammalian cells: Involvement of the peroxisomal targeting signal. J. Cell Biol..

[56] Titorenko V.I., Nicaud J.M., Wang H., Chan H., Rachubinski R.A. (2002). Acyl-CoA oxidase is imported as a heteropentameric, cofactor-containing complex into peroxisomes of Yarrowia lipolytica. J. Cell Biol..

[57] Glover J.R., Andrews D.W., Rachubinski R.A. (1994). Saccharomyces cerevisiae peroxisomal thiolase is imported as a dimer. Proc. Natl. Acad. Sci. U. S. A..

[58] Yang J., Pieuchot L., Jedd G. (2018). Artificial import substrates reveal an omnivorous peroxisomal importomer. Traffic.

[59] Walton P.A., Hill P.E., Subramani S. (1995). Import of stably folded proteins into peroxisomes. Mol. Biol. Cell.

[60] Green E.R., Mecsas J. (2016). Bacterial secretion systems: an overview. Microbiol. Spectr..

[61] Korotkov K.V., Sandkvist M., Hol W.G. (2012). The type II secretion system: biogenesis, molecular architecture and mechanism. Nat. Rev. Microbiol..

[62] Naskar S., Hohl M., Tassinari M., Low H.H. (2021). The structure and mechanism of the bacterial type II secretion system. Mol. Microbiol..

[63] Hirst T.R., Holmgren J. (1987). Conformation of protein secreted across bacterial outer membranes: a study of enterotoxin translocation from Vibrio cholerae. Proc. Natl. Acad. Sci. U. S. A..

[64] Pugsley A.P. (1992). Translocation of a folded protein across the outer membrane in Escherichia coli. Proc. Natl. Acad. Sci. U. S. A..

[65] Bortoli-German I., Brun E., Py B., Chippaux M., Barras F. (1994). Periplasmic disulphide bond formation is essential for cellulase secretion by the plant pathogen *Erwinia chrysanthemi*. Mol. Microbiol..

[66] Shevchik V.E., Bortoli-German I., Robert-Baudouy J., Robinet S., Barras F., Condemine G. (1995). Differential effect of dsbA and dsbC mutations on extracellular enzyme secretion in *Erwinia chrysanthemi*. Mol. Microbiol..

[67] Chapon V., Simpson H.D., Morelli X., Brun E., Barras F. (2000). Alteration of a single tryptophan residue of the cellulose-binding domain blocks secretion of the *Erwinia chrysanthemi* Cel5 cellulase (ex-EGZ) *via* the type II system. J. Mol. Biol..

[68] Hardie K.R., Schulze A., Parker M.W., Buckley J.T. (1995). Vibrio spp. secrete proaerolysin as a folded dimer without the need for disulphide bond formation. Mol. Microbiol..

[69] Gorasia D.G., Veith P.D., Reynolds E.C. (2020). The type IX secretion system: advances in structure, function and organisation. Microorganisms.

[70] Lasica A.M., Ksiazek M., Madej M., Potempa J. (2017). The type IX secretion system (T9SS): highlights and recent insights into its structure and function. Front. Cell Infect Microbiol.

[71] Shoji M., Sato K., Yukitake H., Kondo Y., Narita Y., Kadowaki T. (2011). Por secretion system-dependent secretion and glycosylation of *Porphyromonas gingivalis* hemin-binding protein 35. PLoS One.

[72] Kulkarni S.S., Zhu Y., Brendel C.J., McBride M.J. (2017). Diverse C-terminal sequences involved in flavobacterium johnsoniae protein secretion. J. Bacteriol..

[73] Champion P.A., Stanley S.A., Champion M.M., Brown E.J., Cox J.S. (2006). C-terminal signal sequence promotes virulence factor secretion in *Mycobacterium tuberculosis*. Science.

[74] Daleke M.H., Ummels R., Bawono P., Heringa J., Vandenbroucke-Grauls C.M., Luirink J. (2012). General secretion signal for the mycobacterial type VII secretion pathway. Proc. Natl. Acad. Sci. U. S. A..

[75] Rosenberg O.S., Dovala D., Li X., Connolly L., Bendebury A., Finer-Moore J. (2015). Substrates control multimerization and activation of the multi-domain ATPase motor of type VII secretion. Cell.

[76] Wang S., Zhou K., Yang X., Zhang B., Zhao Y., Xiao Y. (2020). Structural insights into substrate recognition by the type VII secretion system. Protein Cell.

[77] Krantz B.A., Finkelstein A., Collier R.J. (2006). Protein translocation through the anthrax toxin transmembrane pore is driven by a proton gradient. J. Mol. Biol..

[78] Senzel L., Huynh P.D., Jakes K.S., Collier R.J., Finkelstein A. (1998). The diphtheria toxin channel-forming T domain translocates its own NH2-terminal region across planar bilayers. J. Gen. Physiol..

[79] Blaustein R.O., Koehler T.M., Collier R.J., Finkelstein A. (1989). Anthrax toxin: channel-forming activity of protective antigen in planar phospholipid bilayers. Proc. Natl. Acad. Sci. U. S. A..

[80] Knapp O., Benz R., Popoff M.R. (2016). Pore-forming activity of clostridial binary toxins. Biochim. Biophys. Acta.

[81] Anderson D.M., Sheedlo M.J., Jensen J.L., Lacy D.B. (2020). Structural insights into the transition of *Clostridioides difficile* binary toxin from prepore to pore. Nat. Microbiol..

[82] Kakimoto S., Hamada T., Komatsu Y., Takagi M., Tanabe T., Azuma H. (2009). The conjugation of diphtheria toxin T domain to poly(ethylenimine) based vectors for enhanced endosomal escape during gene transfection. Biomaterials.

[83] Arnold A.E., Smith L.J., Beilhartz G.L., Bahlmann L.C., Jameson E., Melnyk R.A. (2020). Attenuated diphtheria toxin mediates siRNA delivery. Sci. Adv..

[84] Turco M.M., Sousa M.C. (2014). The structure and specificity of the type III secretion system effector NleC suggest a DNA mimicry mechanism of substrate recognition. Biochemistry.

[85] Maier O., Galan D.L., Wodrich H., Wiethoff C.M. (2010). An N-terminal domain of adenovirus protein VI fragments membranes by inducing positive membrane curvature. Virology.

[86] Ivanovic T., Agosto M.A., Zhang L., Chandran K., Harrison S.C., Nibert M.L. (2008). Peptides released from reovirus outer capsid form membrane pores that recruit virus particles. EMBO J..

[87] Girod A., Wobus C.E., Zadori Z., Ried M., Leike K., Tijssen P. (2002). The VP1 capsid protein of adeno-associated virus type 2 is carrying a phospholipase A2 domain required for virus infectivity. J. Gen. Virol..

[88] Wickham T.J., Mathias P., Cheresh D.A., Nemerow G.R. (1993). Integrins alpha v beta 3 and alpha v beta 5 promote adenovirus internalization but not virus attachment. Cell.

[89] Day P.M., Lowy D.R., Schiller J.T. (2003). Papillomaviruses infect cells *via* a clathrin-dependent pathway. Virology.

[90] Pelkmans L., Kartenbeck J., Helenius A. (2001). Caveolar endocytosis of simian virus 40 reveals a new two-step vesicular-transport pathway to the ER. Nat. Cell Biol..

[91] Schelhaas M., Malmstrom J., Pelkmans L., Haugstetter J., Ellgaard L., Grunewald K. (2007). Simian Virus 40 depends on ER protein folding and quality control factors for entry into host cells. Cell.

[92] Hariton-Gazal E., Rosenbluh J., Graessmann A., Gilon C., Loyter A. (2003). Direct translocation of histone molecules across cell membranes. J. Cell Sci..

[93] Gallegos S., Pacheco C., Peters C., Opazo C.M., Aguayo L.G. (2015). Features of alpha-synuclein that could explain the progression and irreversibility of Parkinson's disease. Front. Neurosci..

[94] Hopkins B.D., Fine B., Steinbach N., Dendy M., Rapp Z., Shaw J. (2013). A secreted PTEN phosphatase that enters cells to alter signaling and survival. Science.

[95] Rivadeneyra-Espinoza L., Ruiz-Arguelles A. (2006). Cell-penetrating anti-native DNA antibodies trigger apoptosis through both the neglect and programmed pathways. J. Autoimmun..

[96] Zahaf N.I., Schmidt G. (2017). Bacterial toxins for cancer therapy. Toxins (Basel).

[97] Schwarze S.R., Ho A., Vocero-Akbani A., Dowdy S.F. (1999). *In vivo* protein transduction: delivery of a biologically active protein into the mouse. Science.

[98] Chen K., Pei D. (2020). Engineering cell-permeable proteins through insertion of cell-penetrating motifs into surface loops. ACS Chem. Biol..

[99] Kim J.S., Choi D.K., Shin J.Y., Shin S.M., Park S.W., Cho H.S. (2016). Endosomal acidic pH-induced conformational changes of a cytosol-penetrating antibody mediate endosomal escape. J. Control Release.

[100] Hurtley S.M., Helenius A. (1989). Protein oligomerization in the endoplasmic reticulum. Annu. Rev. Cell Biol..

[101] Carvalho P., Goder V., Rapoport T.A. (2006). Distinct ubiquitin-ligase complexes define convergent pathways for the degradation of ER proteins. Cell.

[102] Denic V., Quan E.M., Weissman J.S. (2006). A luminal surveillance complex that selects misfolded glycoproteins for ER-associated degradation. Cell.

[103] Gauss R., Jarosch E., Sommer T., Hirsch C. (2006). A complex of Yos9p and the HRD ligase integrates endoplasmic reticulum quality control into the degradation machinery. Nat. Cell Biol..

[104] Hebert D.N., Molinari M. (2007). In and out of the ER: protein folding, quality control, degradation, and related human diseases. Physiol. Rev..

[105] Nowakowska-Golacka J., Sominka H., Sowa-Rogozinska N., Slominska-Wojewodzka M. (2019). Toxins utilize the endoplasmic reticulum-associated protein degradation pathway in their intoxication process. Int. J. Mol. Sci..

[106] Tsai B., Rodighiero C., Lencer W.I., Rapoport T.A. (2001). Protein disulfide isomerase acts as a redox-dependent chaperone to unfold cholera toxin. Cell.

[107] Daniels R., Rusan N.M., Wadsworth P., Hebert D.N. (2006). SV40 VP2 and VP3 insertion into ER membranes is controlled by the capsid protein VP1: implications for DNA translocation out of the ER. Mol. Cell.

[108] Rainey-Barger E.K., Magnuson B., Tsai B. (2007). A chaperone-activated nonenveloped virus perforates the physiologically relevant endoplasmic reticulum membrane. J. Virol..

[109] Magnuson B., Rainey E.K., Benjamin T., Baryshev M., Mkrtchian S., Tsai B. (2005). ERp29 triggers a conformational change in polyomavirus to stimulate membrane binding. Mol. Cell.

[110] Bagchi P., Inoue T., Tsai B. (2016). EMC1-dependent stabilization drives membrane penetration of a partially destabilized non-enveloped virus. Elife.

[111] Inoue T., Tsai B. (2011). A large and intact viral particle penetrates the endoplasmic reticulum membrane to reach the cytosol. PLoS Pathog..

[112] Wagener N., Ackermann M., Funes S., Neupert W. (2011). A pathway of protein translocation in mitochondria mediated by the AAA-ATPase Bcs1. Mol. Cell.

[113] Nivaskumar M., Francetic O. (2014). Type II secretion system: a magic beanstalk or a protein escalator. Biochim. Biophys. Acta.

[114] Reichow S.L., Korotkov K.V., Hol W.G., Gonen T. (2010). Structure of the cholera toxin secretion channel in its closed state. Nat. Struct. Mol. Biol..

[115] Korotkov K.V., Gonen T., Hol W.G. (2011). Secretins: dynamic channels for protein transport across membranes. Trends Biochem. Sci..

[116] Yan Z., Yin M., Xu D., Zhu Y., Li X. (2017). Structural insights into the secretin translocation channel in the type II secretion system. Nat. Struct. Mol. Biol..

[117] Shevchik V.E., Robert-Baudouy J., Condemine G. (1997). Specific interaction between OutD, an *Erwinia chrysanthemi* outer membrane protein of the general secretory pathway, and secreted proteins. EMBO J..

[118] Lauber F., Deme J.C., Lea S.M., Berks B.C. (2018). Type 9 secretion system structures reveal a new protein transport mechanism. Nature.

[119] Seers C.A., Slakeski N., Veith P.D., Nikolof T., Chen Y.Y., Dashper S.G. (2006). The RgpB C-terminal domain has a role in attachment of RgpB to the outer membrane and belongs to a novel C-terminal-domain family found in *Porphyromonas gingivalis*. J. Bacteriol..

[120] Veith P.D., Nor Muhammad N.A., Dashper S.G., Likic V.A., Gorasia D.G., Chen D. (2013). Protein substrates of a novel secretion system are numerous in the Bacteroidetes phylum and have in common a cleavable C-terminal secretion signal, extensive post-translational modification, and cell-surface attachment. J. Proteome Res..

[121] Zeitler M., Steringer J.P., Muller H.M., Mayer M.P., Nickel W. (2015). HIV-Tat protein forms phosphoinositide-dependent membrane pores implicated in unconventional protein secretion. J. Biol. Chem..

[122] Monteleone M., Stanley A.C., Chen K.W., Brown D.L., Bezbradica J.S., von Pein J.B. (2018). Interleukin-1beta maturation triggers its relocation to the plasma membrane for gasdermin-D-dependent and -independent secretion. Cell Rep..

[123] Xia S., Zhang Z., Magupalli V.G., Pablo J.L., Dong Y., Vora S.M. (2021). Gasdermin D pore structure reveals preferential release of mature interleukin-1. Nature.

[124] Ding J., Wang K., Liu W., She Y., Sun Q., Shi J. (2016). Pore-forming activity and structural autoinhibition of the gasdermin family. Nature.

[125] Santa Cruz Garcia A.B., Schnur K.P., Malik A.B., Mo G.C.H. (2022). Gasdermin D pores are dynamically regulated by local phosphoinositide circuitry. Nat. Commun..

[126] Meinecke M., Cizmowski C., Schliebs W., Kruger V., Beck S., Wagner R. (2010). The peroxisomal importomer constitutes a large and highly dynamic pore. Nat. Cell Biol..

[127] Ma C., Schumann U., Rayapuram N., Subramani S. (2009). The peroxisomal matrix import of Pex8p requires only PTS receptors and Pex14p. Mol. Biol. Cell.

[128] Walter T., Erdmann R. (2019). Current advances in protein import into peroxisomes. Protein J..

[129] Kater L., Wagener N., Berninghausen O., Becker T., Neupert W., Beckmann R. (2020). Structure of the Bcs1 AAA-ATPase suggests an airlock-like translocation mechanism for folded proteins. Nat. Struct. Mol. Biol..

[130] Tang W.K., Borgnia M.J., Hsu A.L., Esser L., Fox T., de Val N. (2020). Structures of AAA protein translocase Bcs1 suggest translocation mechanism of a folded protein. Nat. Struct. Mol. Biol..

[131] Gordon V.M., Leppla S.H., Hewlett E.L. (1988). Inhibitors of receptor-mediated endocytosis block the entry of Bacillus anthracis adenylate cyclase toxin but not that of Bordetella pertussis adenylate cyclase toxin. Infect. Immun..

[132] Veneziano R., Rossi C., Chenal A., Devoisselle J.M., Ladant D., Chopineau J. (2013). Bordetella pertussis adenylate cyclase toxin translocation across a tethered lipid bilayer. Proc. Natl. Acad. Sci. U. S. A..

[133] Osickova A., Masin J., Fayolle C., Krusek J., Basler M., Pospisilova E. (2010). Adenylate cyclase toxin translocates across target cell membrane without forming a pore. Mol. Microbiol..

[134] Subrini O., Sotomayor-Perez A.C., Hessel A., Spiaczka-Karst J., Selwa E., Sapay N. (2013). Characterization of a membrane-active peptide from the Bordetella pertussis CyaA toxin. J. Biol. Chem..

[135] Moyer C.L., Nemerow G.R. (2011). Viral weapons of membrane destruction: variable modes of membrane penetration by non-enveloped viruses. Curr. Opin. Virol..

[136] Degors I.M.S., Wang C., Rehman Z.U., Zuhorn I.S. (2019). Carriers break barriers in drug delivery: endocytosis and endosomal escape of gene delivery vectors. Acc. Chem. Res..

[137] Wu X., Rapoport T.A. (2021). Translocation of proteins through a distorted lipid bilayer. Trends Cell Biol..

[138] Walczak C.P., Ravindran M.S., Inoue T., Tsai B. (2014). A cytosolic chaperone complexes with dynamic membrane J-proteins and mobilizes a nonenveloped virus out of the endoplasmic reticulum. PLoS Pathog..

[139] Ravindran M.S., Bagchi P., Inoue T., Tsai B. (2015). A non-enveloped virus hijacks host disaggregation machinery to translocate across the endoplasmic reticulum membrane. PLoS Pathog..

[140] Dupzyk A., Williams J.M., Bagchi P., Inoue T., Tsai B. (2017). SGTA-dependent regulation of Hsc70 promotes cytosol entry of simian virus 40 from the endoplasmic reticulum. J. Virol..

[141] Dupzyk A., Tsai B. (2018). Bag2 Is a component of a cytosolic extraction machinery that promotes membrane penetration of a nonenveloped virus. J. Virol..

[142] Bogsch E.G., Sargent F., Stanley N.R., Berks B.C., Robinson C., Palmer T. (1998). An essential component of a novel bacterial protein export system with homologues in plastids and mitochondria. J. Biol. Chem..

[143] Sargent F., Stanley N.R., Berks B.C., Palmer T. (1999). Sec-independent protein translocation in *Escherichia coli.* A distinct and pivotal role for the TatB protein. J. Biol. Chem..

[144] Gohlke U., Pullan L., McDevitt C.A., Porcelli I., de Leeuw E., Palmer T. (2005). The TatA component of the twin-arginine protein transport system forms channel complexes of variable diameter. Proc. Natl. Acad. Sci. U. S. A..

[145] Rodriguez F., Rouse S.L., Tait C.E., Harmer J., De Riso A., Timmel C.R. (2013). Structural model for the protein-translocating element of the twin-arginine transport system. Proc. Natl. Acad. Sci. U. S. A..

[146] Bolhuis A., Mathers J.E., Thomas J.D., Barrett C.M., Robinson C. (2001). TatB and TatC form a functional and structural unit of the twin-arginine translocase from *Escherichia coli*. J. Biol. Chem..

[147] Cline K., Mori H. (2001). Thylakoid DeltapH-dependent precursor proteins bind to a cpTatC-Hcf106 complex before Tha4-dependent transport. J. Cell Biol..

[148] Alami M., Luke I., Deitermann S., Eisner G., Koch H.G., Brunner J. (2003). Differential interactions between a twin-arginine signal peptide and its translocase in. Escherichia Coli. Mol. Cell.

[149] de Leeuw E., Granjon T., Porcelli I., Alami M., Carr S.B., Muller M. (2002). Oligomeric properties and signal peptide binding by *Escherichia coli* Tat protein transport complexes. J. Mol. Biol..

[150] Leake M.C., Greene N.P., Godun R.M., Granjon T., Buchanan G., Chen S. (2008). Variable stoichiometry of the TatA component of the twin-arginine protein transport system observed by *in vivo* single-molecule imaging. Proc. Natl. Acad. Sci. U. S. A..

[151] Mori H., Cline K. (2002). A twin arginine signal peptide and the pH gradient trigger reversible assembly of the thylakoid [Delta]pH/Tat translocase. J. Cell Biol..

[152] Dabney-Smith C., Cline K. (2009). Clustering of C-terminal stromal domains of Tha4 homo-oligomers during translocation by the Tat protein transport system. Mol. Biol. Cell.

[153] DeLisa M.P., Lee P., Palmer T., Georgiou G. (2004). Phage shock protein PspA of *Escherichia coli* relieves saturation of protein export *via* the Tat pathway. J. Bacteriol..

[154] Lo S.M., Theg S.M. (2012). Role of vesicle-inducing protein in plastids 1 in cpTat transport at the thylakoid. Plant J..

[155] Mehner D., Osadnik H., Lunsdorf H., Bruser T. (2012). The Tat system for membrane translocation of folded proteins recruits the membrane-stabilizing Psp machinery in *Escherichia coli*. J. Biol. Chem..

[156] Bernal-Cabas M., Miethke M., Antelo-Varela M., Aguilar Suarez R., Neef J., Schon L. (2020). Functional association of the stress-responsive LiaH protein and the minimal TatAyCy protein translocase in *Bacillus subtilis*. Biochim. Biophys. Acta Mol. Cell Res..

[157] Hou B., Heidrich E.S., Mehner-Breitfeld D., Bruser T. (2018). The TatA component of the twin-arginine translocation system locally weakens the cytoplasmic membrane of *Escherichia coli* upon protein substrate binding. J. Biol. Chem..

[158] MacKenzie A., Wilson H.L., Kiss-Toth E., Dower S.K., North R.A., Surprenant A. (2001). Rapid secretion of interleukin-1beta by microvesicle shedding. Immunity.

[159] Lindemann S., Tolley N.D., Dixon D.A., McIntyre T.M., Prescott S.M., Zimmerman G.A. (2001). Activated platelets mediate inflammatory signaling by regulated interleukin 1beta synthesis. J. Cell Biol..

[160] Pizzirani C., Ferrari D., Chiozzi P., Adinolfi E., Sandona D., Savaglio E. (2007). Stimulation of P2 receptors causes release of IL-1beta-loaded microvesicles from human dendritic cells. Blood.

[161] Mouasni S., Gonzalez V., Schmitt A., Bennana E., Guillonneau F., Mistou S. (2019). The classical NLRP3 inflammasome controls FADD unconventional secretion through microvesicle shedding. Cell Death Dis..

[162] Kreger B.T., Dougherty A.L., Greene K.S., Cerione R.A., Antonyak M.A. (2016). Microvesicle cargo and function changes upon induction of cellular transformation. J. Biol. Chem..

[163] Kielian M., Jungerwirth S. (1990). Mechanisms of enveloped virus entry into cells. Mol. Biol. Med..

[164] Wang B.Z., Luo L.J., Vunjak-Novakovic G. (2021). RNA and protein delivery by cell-secreted and bioengineered extracellular vesicles. Adv. Healthc. Mater..

[165] Jeppesen D.K., Fenix A.M., Franklin J.L., Higginbotham J.N., Zhang Q., Zimmerman L.J. (2019). Reassessment of exosome composition. Cell.

[166] Hessvik N.P., Llorente A. (2018). Current knowledge on exosome biogenesis and release. Cell Mol. Life Sci..

[167] Choi D.S., Kim D.K., Kim Y.K., Gho Y.S. (2013). Proteomics, transcriptomics and lipidomics of exosomes and ectosomes. Proteomics.

[168] Qu Y., Franchi L., Nunez G., Dubyak G.R. (2007). Nonclassical IL-1 beta secretion stimulated by P2X7 receptors is dependent on inflammasome activation and correlated with exosome release in murine macrophages. J. Immunol..

[169] Thery C., Ostrowski M., Segura E. (2009). Membrane vesicles as conveyors of immune responses. Nat. Rev. Immunol..

[170] Zhang M., Liu L., Lin X., Wang Y., Li Y., Guo Q. (2020). A translocation pathway for vesicle-mediated unconventional protein secretion. Cell.

[171] Moreau K., Luo S., Rubinsztein D.C. (2010). Cytoprotective roles for autophagy. Curr. Opin. Cell Biol..

[172] Klionsky D.J., Eskelinen E.L., Deretic V. (2014). Autophagosomes, phagosomes, autolysosomes, phagolysosomes, autophagolysosomes... wait, I'm confused. Autophagy.

[173] Qian Z., Martyna A., Hard R.L., Wang J., Appiah-Kubi G., Coss C. (2016). Discovery and mechanism of highly efficient cyclic cell-penetrating peptides. Biochemistry.

[174] Sahni A., Qian Z., Pei D. (2020). Cell-penetrating peptides escape the endosome by inducing vesicle budding and collapse. ACS Chem. Biol..

[175] Pei D. (2022). How do biomolecules cross the cell membrane?. Acc. Chem. Res..

[176] Julicher F., Lipowsky R. (1993). Domain-induced budding of vesicles. Phys. Rev. Lett..

[177] Dougherty P.G., Sahni A., Pei D. (2019). Understanding cell penetration of cyclic peptides. Chem. Rev..

[178] Lee M.S., Mullen R.T., Trelease R.N. (1997). Oilseed isocitrate lyases lacking their essential type 1 peroxisomal targeting signal are piggybacked to glyoxysomes. Plant Cell.

[179] Rodrigue A., Chanal A., Beck K., Muller M., Wu L.F. (1999). Co-translocation of a periplasmic enzyme complex by a hitchhiker mechanism through the bacterial tat pathway. J. Biol. Chem..

[180] McNew J.A., Goodman J.M. (1994). An oligomeric protein is imported into peroxisomes *in vivo*. J. Cell Biol..

[181] Elgersma Y., Vos A., van den Berg M., van Roermund C.W., van der Sluijs P., Distel B. (1996). Analysis of the carboxyl-terminal peroxisomal targeting signal 1 in a homologous context in *Saccharomyces cerevisiae*. J. Biol. Chem..

[182] Hudson T.H., Neville D.M. (1985). Quantal entry of diphtheria toxin to the cytosol. J. Biol. Chem..

[183] Dimou E., Cosentino K., Platonova E., Ros U., Sadeghi M., Kashyap P. (2019). Single event visualization of unconventional secretion of FGF2. J. Cell Biol..

[184] Zhang H., Freitas D., Kim H.S., Fabijanic K., Li Z., Chen H. (2018). Identification of distinct nanoparticles and subsets of extracellular vesicles by asymmetric flow field-flow fractionation. Nat. Cell Biol..

[185] Hu Y., Zhao E., Li H., Xia B., Jin C. (2010). Solution NMR structure of the TatA component of the twin-arginine protein transport system from gram-positive bacterium Bacillus subtilis. J. Am. Chem. Soc..

[186] Pettersson P., Patrick J., Jakob M., Jacobs M., Klosgen R.B., Wennmalm S. (2021). Soluble TatA forms oligomers that interact with membranes: structure and insertion studies of a versatile protein transporter. Biochim. Biophys. Acta Biomembr..

[187] Dabney-Smith C., Mori H., Cline K. (2006). Oligomers of Tha4 organize at the thylakoid Tat translocase during protein transport. J. Biol. Chem..

[188] Alcock F., Baker M.A., Greene N.P., Palmer T., Wallace M.I., Berks B.C. (2013). Live cell imaging shows reversible assembly of the TatA component of the twin-arginine protein transport system. Proc. Natl. Acad. Sci. U. S. A..

[189] Teter S.A., Theg S.M. (1998). Energy-transducing thylakoid membranes remain highly impermeable to ions during protein translocation. Proc. Natl. Acad. Sci. U. S. A..

[190] Asher A.H., Theg S.M. (2021). Electrochromic shift supports the membrane destabilization model of Tat-mediated transport and shows ion leakage during Sec transport. Proc. Natl. Acad. Sci. U. S. A..

[191] Ma X., Cline K. (2010). Multiple precursor proteins bind individual Tat receptor complexes and are collectively transported. EMBO J..

[192] Nurizzo D., Halbig D., Sprenger G.A., Baker E.N. (2001). Crystal structures of the precursor form of glucose-fructose oxidoreductase from Zymomonas mobilis and its complexes with bound ligands. Biochemistry.

[193] Gerard F., Cline K. (2006). Efficient twin arginine translocation (Tat) pathway transport of a precursor protein covalently anchored to its initial cpTatC binding site. J. Biol. Chem..

[194] Pérez-Cruz C., Delgado L., López-Iglesias C., Mercade E. (2015). Outer-inner membrane vesicles naturally secreted by Gram-negative pathogenic bacteria. PLoS ONE.

[195] Joly J.C., Wickner W. (1993). The SecA and SecY subunits of translocase are the nearest neighbors of a translocating preprotein, shielding it from phospholipids. EMBO J..

[196] van den Berg B., Clemons W.M., Collinson I., Modis Y., Hartmann E. (2004). X-ray structure of a protein-conducting channel. Nature.

[197] Albiniak A.M., Baglieri J., Robinson C. (2012). Targeting of lumenal proteins across the thylakoid membrane. J. Exp. Bot..

[198] Rabouille C., Malhotra V., Nickel W. (2012). Diversity in unconventional protein secretion. J. Cell Sci..

[199] Eilers M., Schatz G. (1986). Binding of a specific ligand inhibits import of a purified precursor protein into mitochondria. Nature.

[200] Ganesan I., Shi L.X., Labs M., Theg S.M. (2018). Evaluating the functional pore size of chloroplast TOC and TIC protein translocons: import of folded proteins. Plant Cell.

[201] Bowman L., Palmer T. (2021). The type VII secretion system of *Staphylococcus*. Annu. Rev. Microbiol..

